# Gasdermin D is the only Gasdermin that provides protection against acute *Salmonella* gut infection in mice

**DOI:** 10.1073/pnas.2315503120

**Published:** 2023-11-21

**Authors:** Stefan A. Fattinger, Luca Maurer, Petra Geiser, Elliott M. Bernard, Ursina Enz, Suwannee Ganguillet, Ersin Gül, Sanne Kroon, Benjamin Demarco, Vanessa Mack, Markus Furter, Manja Barthel, Pawel Pelczar, Feng Shao, Petr Broz, Mikael E. Sellin, Wolf-Dietrich Hardt

**Affiliations:** ^a^Department of Biology, Institute of Microbiology, ETH Zurich, Zurich 8093, Switzerland; ^b^Department of Medical Biochemistry and Microbiology, Science for Life Laboratory, Uppsala University, Uppsala 75123, Sweden; ^c^Division of Immunology and Molecular Medicine, Department of Molecular and Cell Biology, University of California, Berkeley, CA 94720; ^d^Department of Immunobiology, University of Lausanne, Epalinges 1066, Switzerland; ^e^Center for Transgenic Models, University of Basel, Basel 4002, Switzerland; ^f^National Institute of Biological Sciences, Beijing 102206, China

**Keywords:** immunology, microbiology, pathogen, pyroptosis

## Abstract

Gasdermins (GSDMs) share a common functional domain structure and are best known for their capacity to form membrane pores. These pores are hallmarks of a specific form of cell death called pyroptosis and mediate the secretion of pro-inflammatory cytokines such as interleukin 1β (IL1β) and interleukin 18 (IL18). Thereby, Gasdermins have been implicated in various immune responses against cancer and infectious diseases such as acute *Salmonella* Typhimurium (*S.*Tm) gut infection. However, to date, we lack a comprehensive functional assessment of the different Gasdermins (GSDMA-E) during *S*.Tm infection in vivo. Here, we used epithelium-specific ablation, bone marrow chimeras, and mouse lines lacking individual Gasdermins, combinations of Gasdermins or even all Gasdermins (GSDMA1-3C1-4DE) at once and performed littermate-controlled oral *S*.Tm infections in streptomycin-pretreated mice to investigate the impact of all murine Gasdermins. While GSDMA, C, and E appear dispensable, we show that GSDMD i) restricts *S*.Tm loads in the gut tissue and systemic organs, ii) controls gut inflammation kinetics, and iii) prevents epithelium disruption by 72 h of the infection. Full protection requires GSDMD expression by both bone-marrow-derived lamina propria cells and intestinal epithelial cells (IECs). In vivo experiments as well as 3D-, 2D-, and chimeric enteroid infections further show that infected IEC extrusion proceeds also without GSDMD, but that GSDMD controls the permeabilization and morphology of the extruding IECs, affects extrusion kinetics, and promotes overall mucosal barrier capacity. As such, this work identifies a unique multipronged role of GSDMD among the Gasdermins for mucosal tissue defense against a common enteric pathogen.

## Significance

The host immune response against infection relies on programmed cell death that has recently been shown to involve Gasdermins—a family of membrane-pore-forming proteins. Despite abundant expression of multiple Gasdermins in mammalian gut tissue, we here find using a mouse line lacking all mouse Gasdermins at once that only Gasdermin D provides protection against oral *Salmonella* infection. To accomplish this protection, both gut epithelial cells and classical immune cells employ Gasdermin D to limit bacterial loads in the mucosa, to control inflammation, to prevent epithelial disruption, and to reduce systemic spread of the pathogen. Hence, this study sheds light on the differential impact of Gasdermins in infectious diseases.

Gasdermins make up a protein family including Gasdermin A, B, C, D, and E (GSDMA, GSDMB, GSDMC, GSDMD, and GSDME, respectively) in humans and GSDMA1-3, GSDMC1-4, GSDMD, and GSDME in mice (1). All members share a common functional domain structure, in which an inhibitory C-terminal domain is linked to a membrane pore-forming N-terminal domain (1, 2). Upon cleavage at the linker region, the N-terminal domain is released to form membrane pores (3–6). These pores mediate lytic cell death and release inflammatory mediators, such as interleukin 1β (IL1β), interleukin 18 (IL18), and lipids into the extracellular milieu to alert neighboring cells (7–9). Gasdermins can be activated with different efficiency by the cysteine proteases Caspase-1, -3, -4, -8, and -11 and by serine proteases to execute cellular responses, thus mediating immunity against pathogens and cancer (7, 9–24). However, their individual roles, cell type specificities, and possible redundancies during oral bacterial infections, such as those caused by *Salmonella enterica* Serovar Typhimurium (*S*.Tm), have not been comprehensively explored.

*S*.Tm is a major foodborne pathogen, a prevalent cause of diarrheal disease worldwide (25), and a risk factor for inflammatory bowel diseases (26). As shown in streptomycin-pretreated mice—a commonly used mouse model for human *Salmonella* diarrhea—*S*.Tm frequently invades intestinal epithelial cells (IECs) during the acute gut infection, transmigrates into the underlying lamina propria compartment, and spreads to systemic organs (27, 28). Innate host immune responses against *S*.Tm include the activation of the NAIP/NLRC4 inflammasome, which senses invading *S*.Tm to induce cell death and interleukin release through a mechanism involving Caspase-1 (29). During *S*.Tm infection of streptomycin-pretreated mice, which develop pronounced *Salmonella* enterocolitis, the NAIP/NLRC4 response, particularly in IECs, provides a first line of defense. This reduces *S.*Tm loads locally in the gut tissue as well as restricts pathogen accumulation at systemic sites like the mesenteric lymph nodes (mLN), spleen, and liver (8, 30–38). The IEC’s NAIP/NLRC4 response limits pathogen spread predominantly by swiftly expelling infected IECs into the gut lumen (8, 31, 37, 39). Importantly, epithelial NAIP/NLRC4 not only triggers cell death but also coordinates the detachment of the infected IEC from the epithelium (a process referred to as IEC extrusion), with a concomitant release of the aforementioned inflammatory mediators (8, 31, 37). In studies using bacteria and/or pure NAIP/NLRC4 ligands, it was shown that GSDMD affects the qualitative features of the IEC extrusion process (8, 21, 40). However, how and to which extent epithelial GSDMD contributes to the overall defense against *S*.Tm infection in vivo remains far from clear. Moreover, we have recently shown that a fraction of the extruding IECs feature activated forms of Caspase-3 and -8 (31), which raises the question if other Gasdermins, which can be activated by those caspases, could additionally be involved in IEC extrusion, and thereby contribute to the defense against *S*.Tm. Indeed, recent studies in mice suggested a role for epithelial GSDME in 2,4,6-trinitrobenzenesulfonic acid-induced colitis (41) and for epithelial GSDMC in worm-infected mice (23, 42). Finally, Gasdermins are originally known for their function in phagocytic immune cells. It has been shown that immune cells employ Gasdermins to promote gut inflammation and defense against several gut pathogens in vivo (11, 15, 43–46). These observations suggest that not only epithelial Gasdermins but also Gasdermins expressed by dedicated immune cells may contribute to pathogen restriction during *S*.Tm infection. However, the respective contribution(s) of particular Gasdermins in IECs and immune cells during acute *Salmonella* diarrhea has not yet been systematically addressed.

Here, we have performed a comprehensive assessment of the impact of Gasdermins during oral *S*.Tm infection. Surprisingly, out of all analyzed Gasdermins, only GSDMD appears to significantly protect against the acute *S*.Tm infection, lowering pathogen loads both in the gut mucosal tissue and at systemic organs. We show that epithelial GSDMD impacts how IECs are extruded into the lumen and that GSDMD in IECs and in bone-marrow-derived phagocytes of the lamina propria work together to prevent *S.*Tm spread beyond the intestinal barrier.

## Results

### Mice Deficient in All Gasdermins, or only Gasdermin D, Feature Elevated *S*.Tm CFUs in the Gut Tissue and in Systemic Organs.

**Fig. 1.**
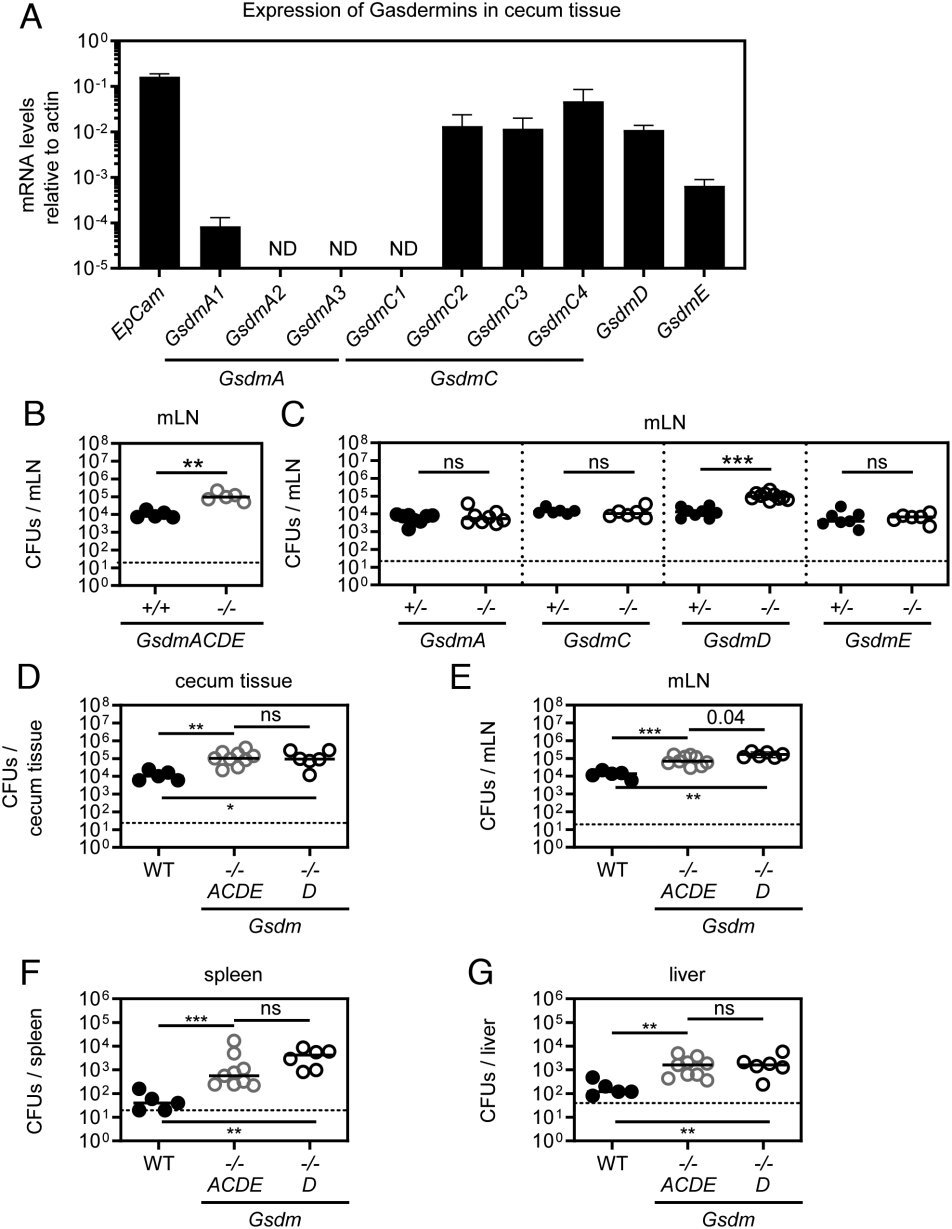
Mice deficient in all Gasdermins, or only Gasdermin D, feature elevated *S*.Tm CFUs in the gut tissue and in systemic organs. (*A*) At 48 h p.i., at least one homologue of each Gasdermin is expressed in the gut tissue. Relative expression levels of individual Gasdermins in the *S*.Tm infected cecum tissue of WT mice as determined by qRT-PCR. ND–Not Detected. (*B*) Gasdermin(s) restrict oral *S*.Tm gut infection. *S*.Tm pathogen loads in the mesenteric lymph nodes of *GsdmACDE^−/−^* mice and WT (*GsdmACDE^+/+^*) mice at 48 h p.i. (*C*) GSDMD is the only Gasdermin with protective function during *S*.Tm gut infection. *S*.Tm pathogen loads in mesenteric lymph nodes of littermate-controlled 48-h infections with GSDMA(A1-A3)-deficient, GSDMC(C1-C4)-deficient, GSDMD-deficient, and GSDME-deficient mice. (*D*–*G*) At 48 h p.i., GSDMD-deficient mice (*GsdmD^−/−^*) phenocopy mice deficient in all Gasdermins (*GsdmACDE^−/−^*) in terms of *S*.Tm pathogen loads. *S*.Tm pathogen loads in (*D*) cecum tissue, (*E*) mesenteric lymph nodes, (*F*) spleen, and (*G*) liver. In *A*, 5 mice were analyzed. Means with SD are indicated. In *B*–*G*, each data point represents one mouse. ≥5 mice per group from ≥2 independent experiments for each comparison. Line at median. The dotted line represents the detection limit. Mann-Whitney *U* test (ns–not significant, *P < 0.05, **P < 0.01, ***P < 0.001).

Gasdermins are activated downstream of cell death pathways and play an important role against pathogens and cancer development (7, 9–24). However, little is known of how they combine to mediate protection against the prototypic gut pathogen *S*.Tm. Therefore, we addressed to what extent and how Gasdermins are involved in the immune response against *S*.Tm infection in mice. Streptomycin-pretreated mice were infected perorally with *S*.Tm (SL1344) for 48 h and RT-qPCR analysis of the cecum tissue—the main invasion site—from Gasdermin-proficient mice revealed that at least one homologue of each Gasdermin is expressed above detection limit (Fig. 1*A*). Using CRISPR/Cas9 genome editing, we generated a knockout mouse line globally lacking all mouse Gasdermins including the multiple homologues for GSDMA and GSDMC (hereafter referred to as *GsdmACDE^−/−^*). In combination with single knockout mice, this multi-Gasdermin-deficient mouse line allows us to address a long-lasting question in the field, namely if redundancies among Gasdermins do exist. To limit microbiota-driven artifacts, these *GsdmACDE^−/−^* mice were co-housed with wild-type (WT) mice for at least two weeks prior to infection. Interestingly, while Gasdermins had no impact on luminal colonization (*SI Appendix*, Fig. S1*A*), *GsdmACDE^−/−^* mice had up to 10-fold elevated *S.*Tm loads in mLN at 48 h post infection (p.i.) suggesting that Gasdermin(s) do restrict *S*.Tm gut infection (Fig. 1*B*). To investigate whether one or several Gasdermins mediate this protection, we performed littermate-controlled infections with mice deficient in individual Gasdermins (GSDMA1-3-, GSDMC1-4-, GSDMD-, or GSDME-deficient mice). In line with the *GsdmACDE^−/−^* mice, luminal *S*.Tm density was similar across genotypes (*SI Appendix*, Fig. S1 *B*–*E*). However, while we did not detect any CFU differences for *GsdmA1-3^−/−^* (hereafter referred to as *GsdmA^−/−^*), *GsdmC1-4^−/−^* (hereafter referred to as *GsdmC^−/−^*), and *GsdmE^−/−^* mice, we did enumerate up to 10-fold more CFUs in the mLN of *GsdmD−/−* mice, suggesting that GSDMD is the critical Gasdermin limiting *S*.Tm loads (Fig. 1*C*). Next, we co-housed WT, *GsdmACDE^−/−^*, and *GsdmD^−/−^* mice and infected them together to verify that the phenotype in *GsdmACDE^−/−^* mice is attributable to GSDMD. In addition, we expanded our analysis and plated cecum tissue as well as other systemic organs such as the spleen and liver. In support of the observations above, we found again higher CFUs for *GsdmACDE^−/−^* and *GsdmD^−/−^* mice in the mLN and also in the cecum tissue and spleen and liver (Fig. 1 *D*–*G* and *SI Appendix*, Fig. S1*F* for luminal colonization). Furthermore, CFU counts appeared similar between *GsdmACDE^−/−^* and *GsdmD^−/−^* animals, indicating that these mice feature a similar phenotype (Fig. 1 *D*–*G*). Of note, none of the other Gasdermin-deficiencies led to a detectable CFU difference in any of the organs (*SI Appendix*, Fig. S1 *G*–*O*). Overall, these data suggest that in contrast to GSDMA1-3, GSDMC1-4, and GSDME, GSDMD reduces *S*.Tm loads in the gut tissue as well as in systemic organs. Thus, GSDMD-deficiency phenocopies *GsdmACDE^−/−^*, which highlights a unique role for GSDMD during acute *S*.Tm gut infection.

### GSDMD Reduces Lamina Propria *S.*Tm Loads and Protects the Gut Tissue Integrity by 72 h of Infection.

**Fig. 2.**
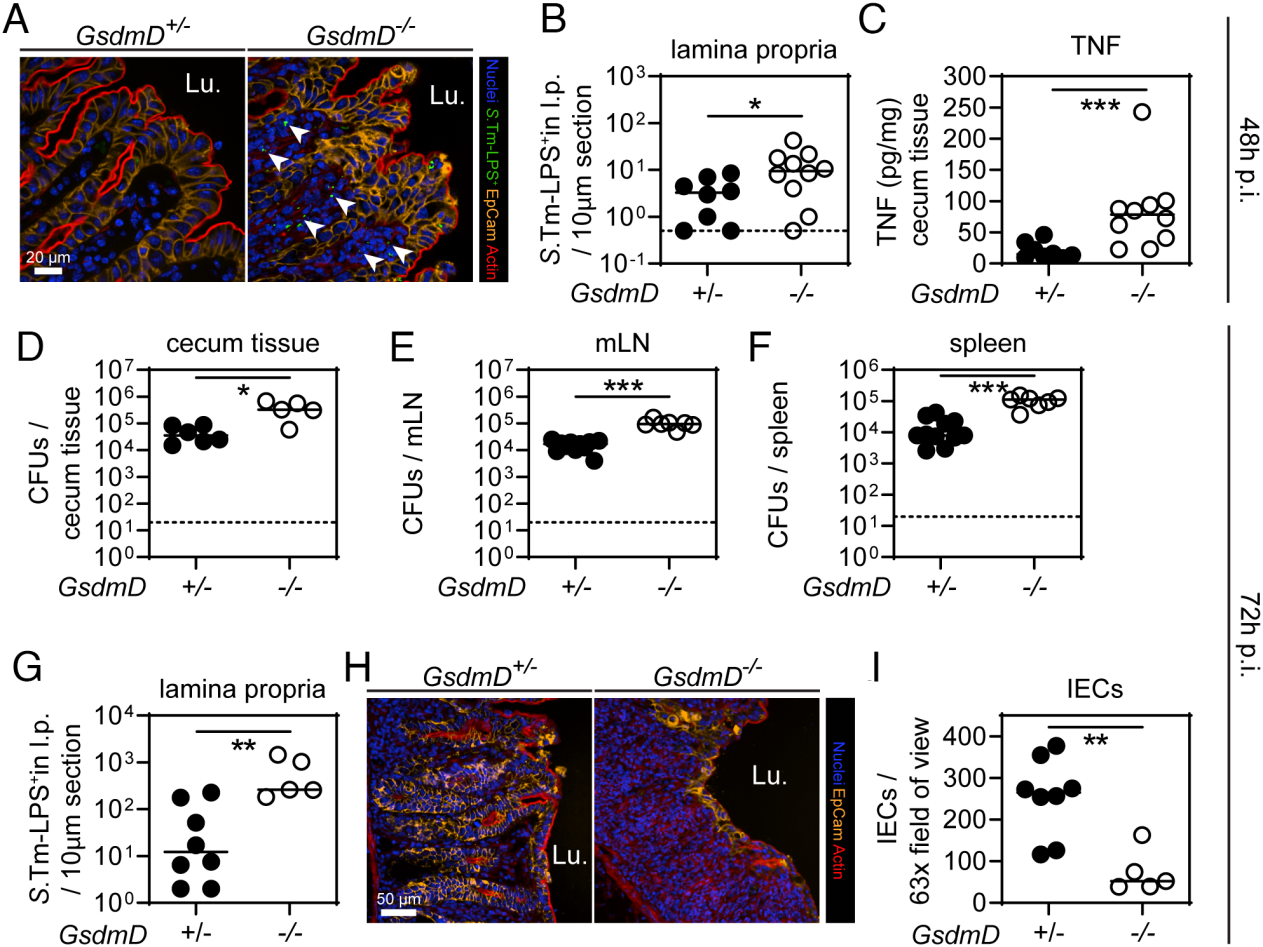
GSDMD reduces lamina propria *S*.Tm loads and protects the gut tissue integrity by 72 h of infection. (*A*–*C*) At 48 h p.i., GSDMD-deficient mice exhibit elevated *S*.Tm pathogen loads in the gut tissue, leading to high levels of TNF compared to heterozygous littermate controls. (*A*) Representative micrographs of cecum tissue sections, stained for *S.*Tm-LPS. Arrowheads indicate *S.*Tm in the lamina propria. Lu. - lumen. (*B*) Microscopy-based quantification of *S.*Tm-LPS^+^ cells in the lamina propria. (*C*) TNF concentrations in cecum tissue. (*D*–*I*) At 72 h p.i., GSDMD deficiency still results in elevated *S*.Tm pathogen loads locally and systemically, and epithelial tissue integrity becomes compromised. *S.*Tm CFU pathogen loads in (*D*) cecum tissue, (*E*) mesenteric lymph nodes, and (*F*) spleen. (*G*) Microscopy-based quantification of *S.*Tm-LPS^+^ cells in the lamina propria. (*H*) Representative micrographs of cecum tissue sections, stained for epithelial marker EpCam. Lu.–lumen. (*I*) Microscopy-based quantification of IECs per 63× field of view. In *B*–*I*, each data point represents one mouse. ≥5 mice per group from ≥2 independent experiments for each comparison. Line at median. The dotted line represents the detection limit. Mann-Whitney *U* test (*P < 0.05, **P < 0.01, ***P < 0.001).

Since the phenotype of *GsdmACDE^−/−^* mice was fully attributable to GSDMD (Fig. 1), we decided to focus our analysis on GSDMD. To exclude any differences at steady-state between *GsdmD^+/−^* and *GsdmD^−/−^* mice in terms of gut inflammation, we analyzed non-infected littermates. In line with previous work (47), the gut mucosa appeared normal in GSDMD-deficient mice, and baseline expression levels of inflammatory mediators were indistinguishable from those from matched littermate controls (*SI Appendix*, Fig. S2 *A*–*C*). GSDMD is activated by Caspase-1 downstream of inflammasomes such as the NAIP/NLRC4 inflammasome (7, 9). Mice deficient in the NAIP/NLRC4 inflammasome accumulate higher *S*.Tm loads in the lamina propria which results in a TNF-driven collapse of the epithelial barrier by days 2–3 after orogastric infection (31). In reminiscence to this NAIP/NLRC4 phenotype, microscopy-based analysis of the cecum tissue at 48 h p.i. revealed elevated *S*.Tm loads in the lamina propria of GSDMD-deficient mice (Fig. 2 *A* and *B*) and cecum TNF levels were significantly increased (Fig. 2*C*). Of note, at this time point of infection, in both *GsdmD^+/−^* and *GsdmD^−/−^* littermates, we measured high levels of the inflammatory marker lipocalin-2 (LCN2) in the feces (*SI Appendix*, Fig. S3*A*).

One day later at 72 h p.i., *S*.Tm CFU loads in the gut tissue as well as at systemic sites were still higher in GSDMD-deficient mice than in the heterozygous littermate controls (Fig. 2 *D*–*F* and *SI Appendix*, Fig. S3 *B* and *C*). Interestingly, *S*.Tm loads remained high in the lamina propria (Fig. 2*G*) and the epithelium became severely disrupted by 72 h p.i. in *GsdmD^−/−^* mice, but not the GSDMD-proficient controls (Fig. 2 *H* and *I*). Accordingly, we observed significantly reduced numbers of IECs and more epithelial gaps than in the corresponding control animals at this point of the infection (Fig. 2 *H* and *I* and *SI Appendix*, Fig. S3*D*). This appeared remarkably similar to the day 3 infection phenotypes previously seen in NAIP/NLRC4-deficient mice (31). These observations were confirmed by independent experiments using an alternative GSDMD-deficient mouse line (*GsdmD_*fsX*^−/−^*), in which the deficiency is caused by a genetic frameshift instead of a deletion (*SI Appendix*, Fig. S3 *E*–*P*). Overall, these data confirm that GSDMD is protective against *S*.Tm infection at ~48 to 72 h p.i., in partial analogy to NAIP/NLRC4 (8, 31, 37). Both GSDMD and NAIP/NLRC4 limit *S*.Tm loads in the deeper gut mucosal tissue, as well as in systemic organs, and prevent the loss of epithelial barrier integrity by 48 to 72 h of infection.

### Bone-Marrow-Derived Cells Employ GSDMD to Restrict *S*.Tm Tissue Loads.

**Fig. 3.**
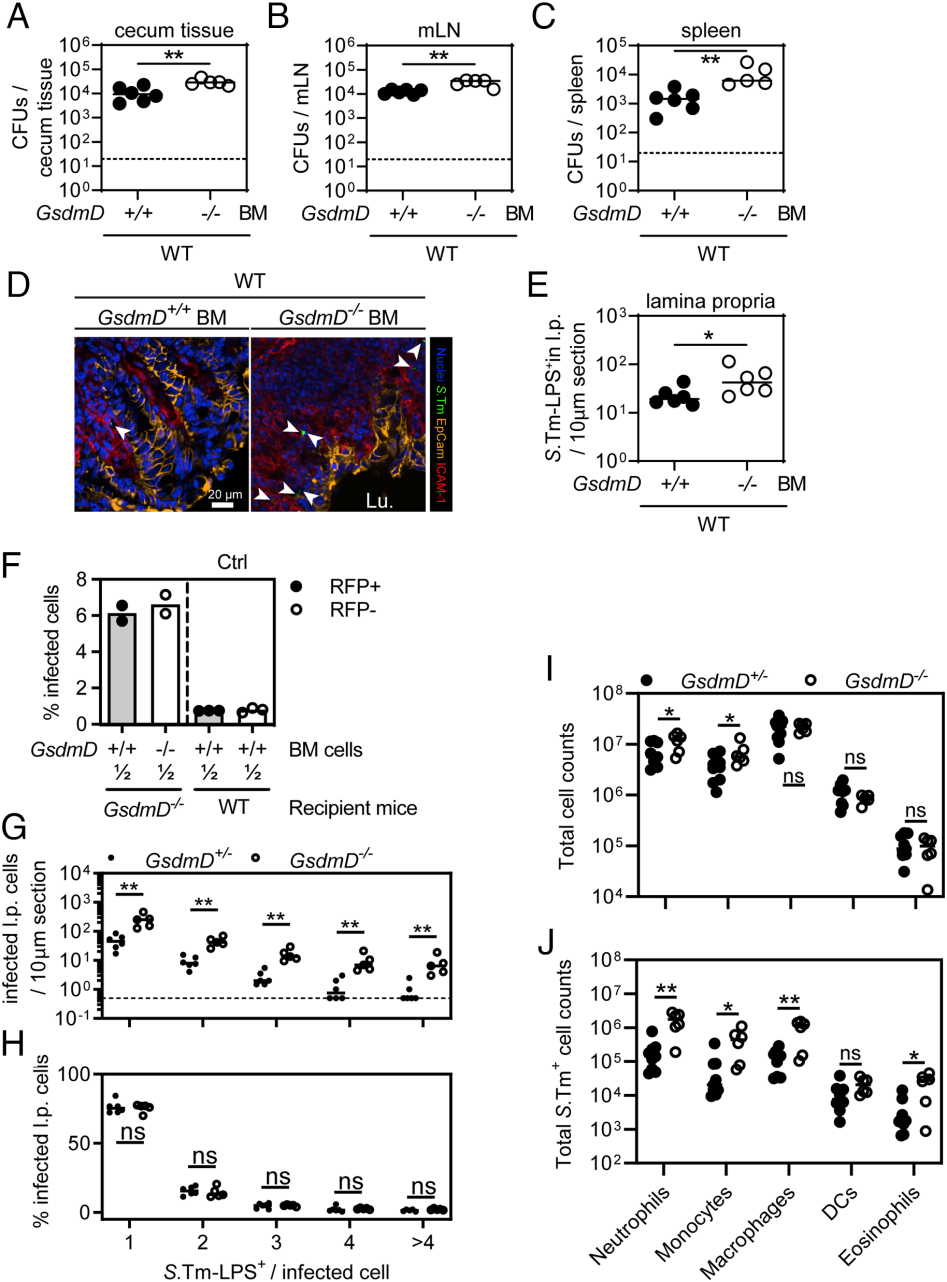
Bone-marrow-derived cells employ GSDMD to restrict *S*.Tm tissue loads. (*A*–*E*) Transfer of *GsdmD^−/−^* bone marrow (BM) cells results in elevated *S*.Tm pathogen loads locally and systemically at 72 h p.i. *S*.Tm pathogen loads in (*A*) cecum tissue, (*B*) mesenteric lymph nodes, and (*C*) spleen. (*D*) Representative micrographs of cecum tissue sections, stained for *S.*Tm-LPS. Arrowheads indicate *S.*Tm in the lamina propria. Lu.–lumen. (*E*) Microscopy-based quantification of *S.*Tm-LPS^+^ cells in the lamina propria. (*F*) *S*.Tm infection of mixed BM chimeras with a 1:1 ratio of either RFP-expressing GSDMD-proficient cells and RFP-non-expressing GSDMD-deficient cells or WT RFP-expressing and non-expressing cells as a control. Percentage of *S*.Tm-LPS^+^ cells determined by flow cytometry. (*G* and *H*) GSDMD deficiency leads to a general increase of infected lamina propria cells. Fluorescence microscopy–based quantification of *S*.Tm-LPS^+^ lamina propria cells at 72 h p.i. (*G*) Microscopy-based quantification of *S.*Tm-LPS^+^ cells in the lamina propria grouped by the number of *S*.Tm-LPS^+^ per cell. (*H*) Relative percentage of the quantification in *G*. (*I* and *J*) Lamina propria cells more frequently harbor *S*.Tm in GSDMD-deficient mice. Flow cytometry analysis of lamina propria cells from 72 h infected *GsdmD^+/−^* and *GsdmD^−/−^* littermates. (*I*) Total cell population sizes in the lamina propria. (*J*) Total *S*.Tm-LPS^+^ cell numbers in the lamina propria. In *A*–*C* and *E*–*J*, each data point represents one mouse. Data are combined from ≥2 independent experiments for each comparison except for *F*, where only one representative experiment is shown out of 2. Line at median. The dotted line represents the detection limit. Mann-Whitney *U* test (ns–not significant, *P < 0.05, **P < 0.01).

Since GSDMD is known for the induction of pyroptosis in bone marrow (BM)-derived macrophages and we observed elevated *S*.Tm loads in the lamina propria of GSDMD-deficient mice, we addressed whether GSDMD in BM-derived cells of the lamina propria restricts *S*.Tm in vivo. WT mice were gamma-irradiated and reconstituted with BM from either WT (CD45.1^+^) or GSDMD-deficient donors, which resulted in >92% transfer efficiency (*SI Appendix*, Fig. S4*A*). When infected, both groups exhibited similar luminal *S*.Tm colonization (*SI Appendix*, Fig. S4*B*). However, *GsdmD^−/−^* BM recipients harbored significantly elevated *S*.Tm loads in the cecum tissue, mLN, and spleen at 72 h p.i. (Fig. 3 *A*–*C*). Moreover, fluorescence microscopy revealed elevated *S*.Tm loads specifically in the lamina propria compartment (Fig. 3 *D* and *E*). This demonstrates that lack of GSDMD exclusively in BM-derived cells is sufficient to observe higher lamina propria *S*.Tm loads after 72 h of infection. Similar observations were made in 48-h infections, or in BM chimeric mice derived from GSDMD-deficient recipients, which were infected for 48 h or 96 h (*SI Appendix*, Fig. S4 *C*, *D*, and *E*–*H*). Accordingly, when *GsdmD^−/−^* mice were infected systemically (intravenous, i.v.), pathogen loads in the spleen and liver were again higher than in the heterozygous littermate controls (*SI Appendix*, Fig. S4 *I* and *J*).

It is well established that membrane pore-formation by GSDMD induces pyroptosis and the release of inflammatory mediators including IL1β or IL18, which could act on neighboring cells to prevent *S*.Tm growth. At 48 h p.i., we did however not detect elevated systemic *S*.Tm loads in IL18-deficient mice (*SI Appendix*, Fig. S4 *K* and *L*). Furthermore, even in the presence of an IL18-depleting antibody, we still enumerated higher systemic pathogen loads in *GsdmD^−/−^* mice compared to heterozygous littermates, suggesting that GSDMD can limit *S*.Tm independently of IL18 (*SI Appendix*, Fig. S4 *M* and *N*). Similar results with limited numbers of mice were obtained upon depleting IL1β instead (*SI Appendix*, Fig. S4 *O* and *P*), suggesting that neither IL18 nor IL1β mediates the GSDMD-dependent *S*.Tm restriction.

To test whether the GSDMD phenotype can be observed on the single cell level, we generated BM chimeras, in which the BM from *GsdmD^−/−^* mice was replaced by a 1:1 mix of RFP-expressing WT (*ActRFP*) and non-fluorescent *GsdmD^−/−^* BM cells (*SI Appendix*, Fig. S5*A* for illustration of the experimental setup). Thereby, we were able to compare GSDMD-deficient and proficient BM-derived cells within the same mouse. As a control, we used WT mice and replaced the BM with a 1:1 mix of RFP-expressing WT (*ActRFP*) and non-fluorescent WT BM cells (*SI Appendix*, Fig. S5*A*). Notably, flow cytometry analysis of lamina propria cells at 72 h p.i. revealed that GSDMD-proficient and -deficient cells in *GsdmD*^−/−^ recipients were infected with a similar frequency considering the difference seen in cells of a GSDMD-proficient mouse (Fig. 3*F*, compare difference of first two bars to the difference to the 3rd and 4th bar, *SI Appendix*, Fig. S5*B*). Hence, in the background of an overall GSDMD-deficient tissue, individual GSDMD-expressing BM-derived cells fail to keep the infection at bay (Fig. 3*F*). Accordingly, *S*.Tm loads were only controlled when no GSDMD-deficient cells were present (Fig. 3*F*). This suggests that rather than GSDMD having a cell-autonomous impact on *S*.Tm loads, GSDMD deficiency appears to negatively affect the ability of the mucosal tissue as a whole to control *S*.Tm loads. In line with these observations, we detected both more single and multiple bacterium-containing lamina propria cells in whole-body *GsdmD^−/−^* mice by fluorescence microscopy (Fig. 3*G* and *SI Appendix*, Fig. S4*Q*), while the GSDMD status did not seem to affect intracellular *S*.Tm growth as judged by the relative proportions of lamina propria cells harboring 1, 2, 3, 4, or >4 bacteria, respectively (Fig. 3*H*).

Next, we sought to address whether the two highly abundant lamina propria cell types, neutrophils and macrophages, employ GSDMD-dependent restriction mechanism(s). This was of interest, since a role of GSDMD has been shown in these cells (43, 48). We depleted neutrophils with anti-Ly6G or macrophages with anti-CSFR1 antibodies and investigated whether *GsdmD^−/−^* mice still featured elevated *S*.Tm organ loads compared to heterozygous littermates. Surprisingly, neither the depletion of neutrophils, nor of macrophages, attenuated the GSDMD-dependent *S*.Tm restriction (*SI Appendix*, Fig. S6 *A*–*G*). Guided by this observation, we explored whether GSDMD deficiency leads to increased *S*.Tm loads in any specific lamina propria cell type. Flow cytometry analysis of lamina propria cells from infected *GsdmD^+/−^* and *GsdmD^−/−^* mice was performed to determine the predominant cell type(s) that harbor *S*.Tm (*SI Appendix*, Fig. S6*H*). Interestingly, while cellular composition was only marginally different between genotypes, multiple cell populations including neutrophils, monocytes, and macrophages (which are most frequent and harbor the highest *S*.Tm loads), but also eosinophils (which are less frequent and harbored lower *S*.Tm loads) all featured elevated fractions of *S.*Tm infected cells when comparing GSDMD-deficient mice to their littermate controls (Fig. 3 *I* and *J*). Taken together, GSDMD deficiency increases *S*.Tm loads across several different types of BM-derived lamina propria cells, particularly in neutrophils, monocytes, macrophages, and eosinophils.

### Epithelial GSDMD Is Dispensable for IEC Extrusion but Affects the Qualitative Features of the Extrusion Process and Promotes Gut Inflammation.

**Fig. 4.**
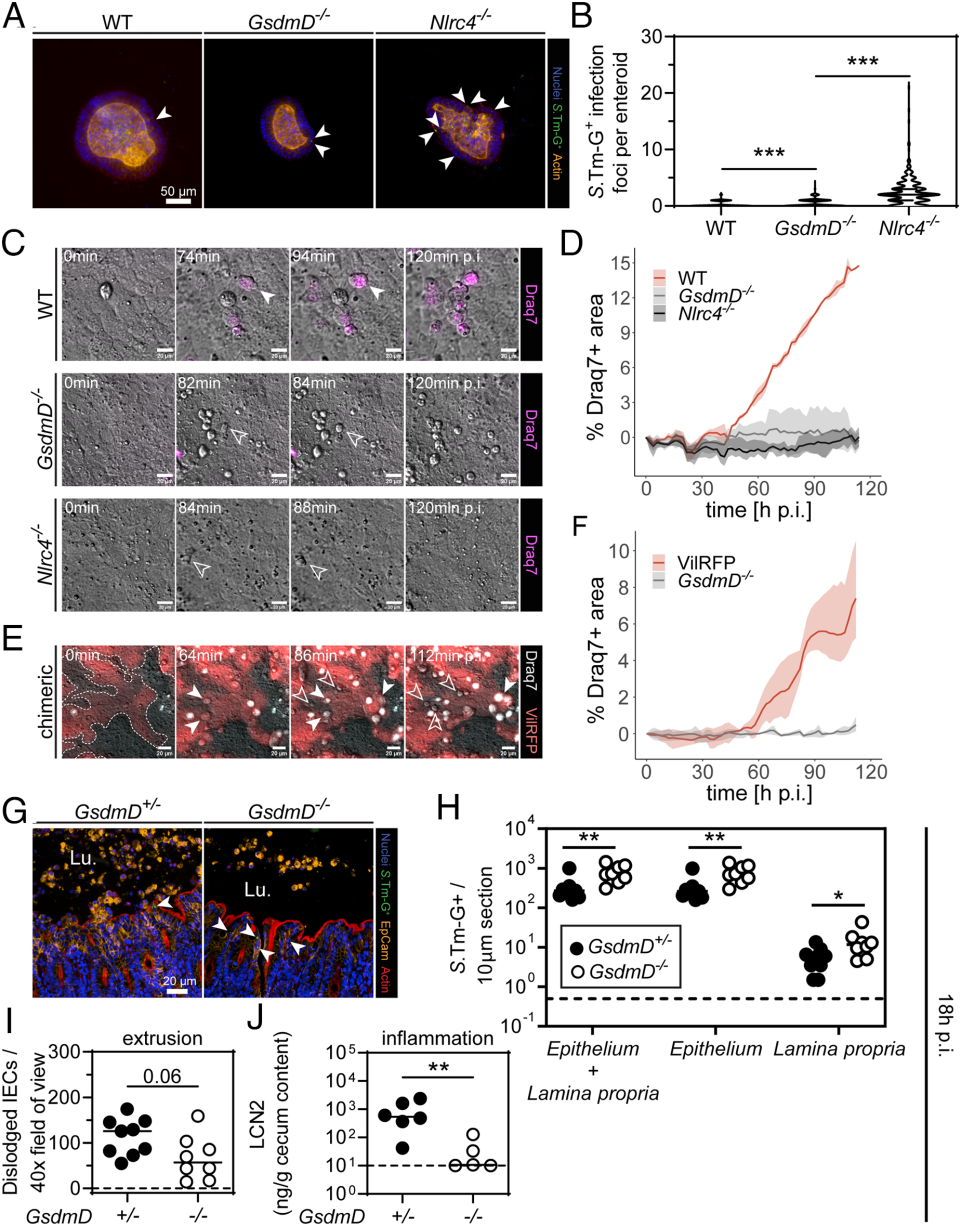
Epithelial GSDMD is dispensable for IEC extrusion but affects the qualitative features of the extrusion process and promotes gut inflammation. (*A* and *B*) Epithelial GSDMD restricts *S*.Tm loads in epithelium but not to the same extent as epithelial NAIP/NLRC4. 3D enteroids were infected in bulk with *S*.Tm harboring a *pssaG-GFP* reporter (renders the bacterium GFP-positive upon host cell entry, *S*.Tm-G^+^) for 4 h. (*A*) Representative micrographs of infected 3D enteroids. Arrowheads indicate *S*.Tm-G^+^. (*B*) Microscopy-based quantification of *S*.Tm-G^+^ in epithelium of enteroids. (*C* and *D*) Epithelial GSDMD impacts qualitative features of extruding infected IECs. Enteroid–monolayers were infected with *S*.Tm in the presence of the membrane-impermeable dye Draq7 to track membrane lysis. (*C*) Representative micrographs of time-lapse microscopy. Filled arrowheads indicate lytic cell death and empty arrowheads indicate non-lytic cell death. (*D*) Quantitative analysis of the Draq7 signal from time-lapse microscopy. (*E* and *F*) GSDMD acts cell-intrinsically on the qualitative features of extruding IECs. Chimeric enteroid–monolayers with GSDMD-proficient (VilRFP) and deficient (*GsdmD^−/−^*) IECs were infected with *S*.Tm in the presence of the membrane-impermeable dye Draq7. (*E*) Representative micrographs of time-lapse microscopy. Filled arrowheads indicate lytic cell death and empty arrowheads indicate non-lytic cell death. (*F*) Quantitative analysis of the Draq7 signal from time-lapse microscopy. (*G*–*J*) 18-h infections with *S*.Tm harboring a *pssaG-GFP* reporter indicate that epithelial GSDMD restricts *S*.Tm in vivo and promotes induction of inflammation. (*G*) Representative micrographs of cecum tissue sections. Arrowheads indicate *S.*Tm-G^+^ in epithelium. Lu.–lumen. (*H*) Microscopy-based quantification of *S.*Tm-G^+^ in mucosal tissue. (*I*) Microscopy-based quantification of dislodged IECs. (*J*) Quantification of inflammation by Lipocalin-2 levels of cecum content. In *A*–*F*, combined results, or representative results from ≥2 replica. In *D* and *F*, mean + range are plotted. In *H*–*J*, each data point represents one mouse. ≥5 mice per group from ≥2 independent experiments for each comparison. Line at median. The dotted line represents the detection limit. Mann-Whitney *U* test (*P < 0.05, **P < 0.01, ***P < 0.001).

The results above demonstrate a restrictive role of GSDMD in lamina propria BM–derived cells. However, it remained incompletely resolved if epithelial GSDMD also contributes significantly to the defense against *S.*Tm, e.g., by cytokine release or by controlling the extrusion of infected IECs. Work in NLRC4-deficient mice had established that epithelial-dependent phenotypes are particularly prominent during the first day of the infection of streptomycin-pretreated mice, or the first few hours in enteroid infection models (8, 31, 37, 40). To tackle this question, we established enteroids from WT and GSDMD-deficient mice, which were infected in-bulk ex vivo with a *S*.Tm reporter strain that turns GFP positive upon host cell invasion (*S*.Tm-G^+^, ref. 49). Notably, in these infected enteroids, we detected cleaved GSDMD to a similar extent as when the enteroids were exposed to the NAIP/NLRC4 agonist RodTox (*SI Appendix*, Fig. S7*A*). Furthermore, using *Nlrc4^−/−^* enteroids as a positive control (31, 40), we quantified *S*.Tm-G^+^ infection foci in more than 70 enteroids per replicate (which represents a sufficient sampling size to obtain statistically valid results; *SI Appendix*, Fig. S7*B*), which revealed that *GsdmD^−/−^* enteroids harbored significantly more intracellular *S*.Tm than the WT controls. The *S*.Tm counts in the *GsdmD^−/−^* enteroids were, however, still much lower in comparison to NLRC4-deficient enteroids (Fig. 4 *A* and *B*).

Given that NLRC4 counteracts *S*.Tm infection by expelling infected IECs into the gut lumen (8, 31, 37, 40), we next sought to address whether and how epithelial GSDMD might contribute to this defense mechanism. To this end, we established enteroid-derived monolayers atop loose hydrogels (50), infected these with *S*.Tm, and followed the IEC extrusion process by differential interference contrast (DIC) and fluorescence live cell microscopy. As expected, NLRC4-deficient monolayers failed to extrude infected IECs (Fig. 4 *C* and *D*). By sharp contrast, in WT and *GsdmD^−/−^* monolayers, we could detect many extruding IECs within 120 min of *S*.Tm infection (Fig. 4 *C* and *D*). Importantly, this process was morphologically distinct between WT and GSDMD-deficient monolayers. IECs extruding from the *GsdmD^−/−^* monolayers appeared round and bright in DIC and remained impermeable for the dye Draq7, while extruding WT IECs were as a rule translucent and eventually all became Draq7 positive (Fig. 4 *C* and *D*). These observations held true even for chimeric monolayers in which we focused on GSDMD-proficient (RFP-labeled) and deficient IECs that were located next to each other, suggesting that this phenomenon is mediated by a cell-intrinsic mechanism (Fig. 4 *E* and *F*). Hence, GSDMD is not required for extrusion per se, but it cell-intrinsically affects the qualitative features of extruding IECs and, in line with previous work (8, 21, 40), the time point of membrane permeabilization.

To investigate the contribution of epithelial GSDMD in vivo, we infected streptomycin-pretreated mice for 18 h, which led to cleaved GSDMD in the cecum tissue (*SI Appendix*, Fig. S7*D*). Of note, one infected mouse out of five did not show a band for cleaved GSDMD (*SI Appendix*, Fig. S7*D*), which might indicate that GSDMD cleavage kinetics differ between mice, that *S*.Tm can block GSDMD cleavage or that it might be able to expedite removal of cleaved GSDMD. Regardless, 80% of the investigated mice show cleaved GSDMD in the cecum tissue by 18 h p.i.. Based on previous work (51), it is well established that up to 18 h p.i., IECs comprise the predominant infected cell type and determine early disease outcome. In line with this, while initial luminal colonization was equal between *GsdmD^−/−^* and *GsdmD^+/−^* littermate controls (*SI Appendix*, Fig. S7*C*), the majority of *S*.Tm-G^+^ in the mucosal tissue was located in IECs (Fig. 4 *G* and *H*, compare *S*.Tm-G^+^ numbers in epithelium vs. lamina propria). Strikingly, already at this earlier time point, we observed increased loads of *S*.Tm-G^+^ in the mucosal tissue of *GsdmD^−/−^* mice, which was mostly attributable to infected IECs (Fig. 4 *G* and *H*). In contrast to the profoundly reduced numbers of expelling infected IECs that we had observed in NLRC4-deficient mice (31, 37), we still observed considerable numbers of dislodged IECs in the GSDMD-deficient mice (Fig. 4*I*). However, GSDMD-deficient mice did feature a trend toward reduced numbers of dislodged IECs, had fewer intraluminal neutrophils, and were significantly less inflamed, as judged by lipocalin-2 levels (Fig. 4 *I* and *J* and *SI Appendix*, Fig. S7 *E* and *F*). In line with previous observations (8), ASC speck formation appeared to be more prevalent in dislodged IECs of *GsdmD^−/−^* mice, even though our analysis has been underpowered to obtain statistically significant evidence for such ASC phenotype (*SI Appendix*, Fig. S7 *G* and *H*). Of note, despite abundant active Caspase-3 in IECs captured in the extrusion process (*SI Appendix*, Fig. S8*A*), a point mutation rendering GSDMD insensitive to inactivation by Caspase-3, did not alter *S*.Tm tissue loads (*SI Appendix*, Fig. S8 *B*–*F*). Moreover, although GSDME can be cleaved by apoptotic Caspases (11, 15–18), we could not observe a similar gut mucosal phenotype in GSDME-deficient mice at 18 h p.i. (*SI Appendix*, Fig. S8 *G*–*K*), not even in a GSDMD-deficient background (*SI Appendix*, Fig. S8 *L* and *P*).

Overall, from these data, we can conclude that although GSDMD is dispensable for IEC extrusion, it can to an extent restrict *S*.Tm in the epithelium (but not by the same magnitude as NAIP/NLRC4). Moreover, GSDMD is essential for prompt cell membrane lysis upon IEC extrusion, which also appears linked to neutrophil recruitment and the initiation of inflammation in the gut mucosa.

### Epithelial GSDMD Contributes to Anti-*S*.Tm Defense in the Mucosa.

**Fig. 5.**
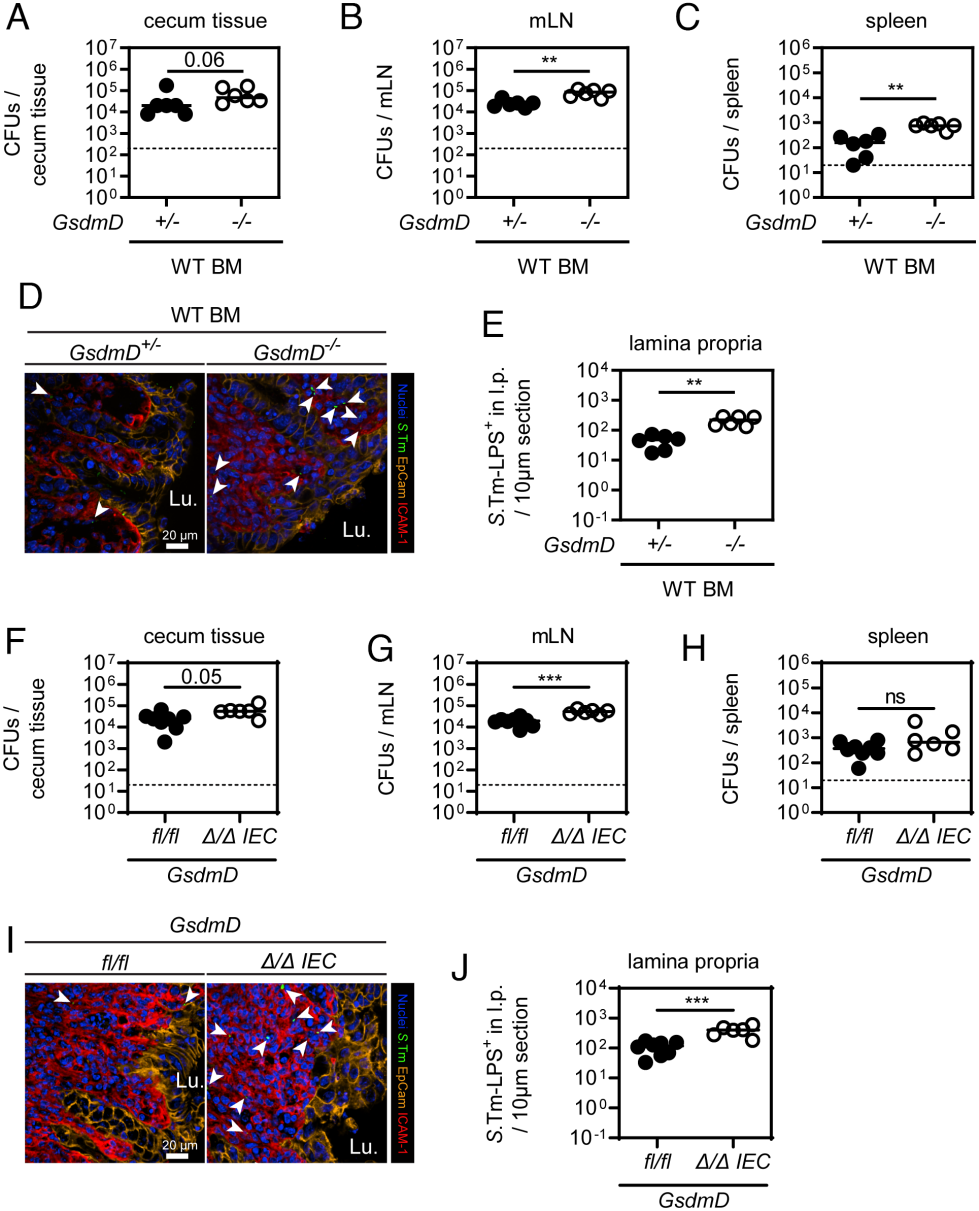
Epithelial GSDMD contributes to anti-S.Tm defense in the mucosa. Epithelial GSDMD contributes to restricting *S*.Tm tissue loads upon oral *S*.Tm infection. (*A*–*E*) Replacement of the BM of *GsdmD^+/−^* and *GsdmD^−/−^* littermates with WT BM cells (CD45.1^+^) does not restore the *S*.Tm pathogen load phenotype of *GsdmD^−/−^* mice locally and systemically at 48 h p.i. *S*.Tm pathogen loads in (*A*) cecum tissue, (*B*) mesenteric lymph nodes, and (*C*) spleen. (*D*) Representative micrographs of cecum tissue sections, stained for *S.*Tm-LPS. Arrowheads indicate *S.*Tm in the lamina propria. Lu.–lumen. (*E*) Microscopy-based quantification of *S.*Tm-LPS^+^ cells in the lamina propria. (*F*–*J*) IEC-specific GSDMD deficiency results in elevated *S*.Tm pathogen loads locally and systemically at 48 h p.i.. *S*.Tm pathogen loads in (*F*) cecum tissue, (*G*) mesenteric lymph nodes, and (*H*) spleen. (*I*) Representative micrographs of cecum tissue sections, stained for *S.*Tm-LPS. Arrowheads indicate *S.*Tm in the lamina propria. Lu.–lumen. (*J*) Microscopy-based quantification of *S.*Tm-LPS^+^ cells in the lamina propria. In *A*–*C*, *E*–*H*, and *J*, each data point represents one mouse. ≥5 mice per group from ≥2 independent experiments for each comparison. Line at median. The dotted line represents the detection limit. Mann-Whitney *U* test (ns–not significant, **P < 0.01, ***P < 0.001).

To directly address whether this epithelial GSDMD phenotype at 18 h p.i. contributes to elevated *S*.Tm loads in cecum tissue, and at systemic sites later during infection, we again generated BM chimeras in which we replaced the BM of *GsdmD^+/−^* and *GsdmD^−/−^* littermates with WT (CD 45.1^+^) BM. Strikingly, despite high transfer efficiency of GSDMD-proficient cells (*SI Appendix*, Fig. S9*A*), we could still detect a tendency toward increased *S*.Tm loads in cecum tissue, as well as significantly elevated loads in mLN, spleen, and liver of GSDMD-deficient recipient mice (Fig. 5 *A*–*C* and *SI Appendix*, Fig. S9 *B* and *C*). Also, in these mice, we enumerated more *S*.Tm-G^+^ in the lamina propria (Fig. 5 *D* and *E*).

To exclude that this difference is attributable to remaining recipient BM-derived cells and to verify the *S*.Tm restriction capacity of epithelial GSDMD, we generated epithelial-specific *GsdmD^−/−^* mice by crossing floxed*-GsdmD* mice with the intestinal-epithelial-specific expressing Cre-recombinase mouse line (*VilCre-*mice). Indeed, at 48 h p.i., while luminal colonization was similar across genotypes (*SI Appendix*, Fig. S9*D*), we observed elevated *S*.Tm loads in the cecum tissue, mLN, spleen and liver from *GsdmD^∆/∆IEC^* compared to *GsdmD^fl/fl^* littermates (Fig. 5 *F*–*H* and *SI Appendix*, Fig. S9*E*). Again, we stained for *S*.Tm-LPS and counted significant more *S*.Tm-LPS^+^ cells in the lamina propria of epithelial-specific GSDMD-deficient animals (Fig. 5 *I* and *J*). This formally demonstrates that epithelial GSDMD contributes to the defense against *S*.Tm in vivo. However, this protection is less pronounced than in the mice with full-body ablation of GSDMD, which is in line with an additional protective function of GSDMD in non-epithelial cells.

Taken together, our combined infection data from BM chimera and mice with cell-type-specific ablation suggest that both, epithelial and lamina propria cell GSDMD, contribute to restricting *S*.Tm tissue loads upon oral *S*.Tm infection.

## Discussion

Gasdermins are key executors of multiple pathogen restriction mechanisms. A limited number of in vivo studies have shown that Gasdermins C, D, and E can reduce organ loads of diverse pathogens, or are involved in immunopathology (11, 22, 23, 41–43, 52, 53). Nevertheless, to date, we still lack a systematic assessment of their potential restrictive role(s) during oral *S*.Tm infection. Our experiments in streptomycin-pretreated mice establish how Gasdermins contribute to *S*.Tm restriction and demonstrate a central function of GSDMD. Moreover, IECs and lamina propria cells both employ GSDMD to restrict *S*.Tm tissue infection and to switch the gut mucosa as a whole toward an anti-*S*.Tm state.

GSDMD is the best-studied Gasdermin to date. GSDMD-deficient mice feature elevated organ pathogen loads during infections with the lung pathogen *Burkholderia cenocepacia* (53). Moreover, GSDMD also plays an important role in protection against gut pathogens such as *Yersinia pseudotuberculosis* and *Citrobacter rodentium* (11, 22, 52). With regard to *S*.Tm infections, it was reported that GSDMD mediates NETosis in neutrophils upon non-canonical Caspase-11 activation by the attenuated *S*.Tm strain *ΔsifA* (48, 54). SifA-deficiency is known to promote egress of *S*.Tm from the *Salmonella* containing vacuole (55), which should enhance pathogen detection in the host cell’s cytosol by Caspase-11. Interestingly, *GsdmD^−/−^* mice intraperitoneally infected with this mutant strain exhibited elevated pathogen loads in the spleen. This appeared to be dependent on the formation of neutrophil extracellular traps (NETs), since DNase I treatment increased spleen pathogen loads in WT mice, but not in GSDMD-deficient animals. Notably, due to the use of a mutant *S*.Tm strain and the intraperitoneal administration (which bypasses the gut tissue invasion steps of the normal infection process), it remained unclear whether this holds true in oral infection with WT *S*.Tm. Here, we demonstrate that GSDMD indeed restricts orally administered WT *S*.Tm not only at the level of the IEC but also in BM-derived lamina propria cells, as well as in systemic organs. However, GSDMD deficiency in a fraction of BM-derived cells appears enough to increase overall tissue *S*.Tm loads and frequency within neutrophils, but also several other immune cells showed elevated *S*.Tm numbers in *GsdmD*^−/−^ mice. This suggests that GSDMD acts globally to restrict *S*.Tm in the mucosal tissue. Since neither the separate depletion of neutrophils, macrophages, or the inflammasome-dependent cytokines IL18 or IL1β significantly impacted the GSDMD phenotype, we speculate that multiple mechanisms may explain this restrictive effect of GSDMD. It is likely that GSDMD in macrophages and neutrophils i) promotes cell death, ii) accelerates mucosal inflammation, and iii) traps *S*.Tm in pore-induced intracellular traps (PITs) and NETs, respectively (54, 56), thereby preventing subsequent re-infections into adjacent host cells. These mechanisms are supported by other previous studies in vivo (43, 48). Nevertheless, it is plausible that additional mechanism(s) may also contribute to the GSDMD-dependent restriction in the lamina propria.

The data from the infection of 3D enteroids, enteroid-derived 2D monolayers (Fig. 4), the early (first 18 h p.i.) infections in *GsdmD*-deficient mice (Fig. 4), the later time point (48 h p.i.) infections with the BM chimeras (Fig. 5) and the epithelial-specific *GsdmD^∆/∆IEC^* experiments (Fig. 5) prove that epithelial GSDMD also contributes to restricting *S*.Tm, most likely during the initial phase of the infection. Importantly, this restriction is much weaker compared to that conferred by epithelial NAIP/NLRC4. This can be explained by the prominent role for NAIP/NLRC4 in driving extrusion of infected IECs, a process which can still be executed in the absence of GSDMD. However, in GSDMD-deficient enteroid–monolayers, we found that the qualitative features of extruding cells appear remarkably different. In line with a recent publication, we show that epithelial GSDMD impacts the time point of membrane lysis during the extrusion process, which also influences how efficiently, and with what kinetics, infected IECs can be removed from the epithelium (21). Furthermore, a delay of cell membrane permeabilization toward much later time points (that is at or after the end of the IEC extrusion process) should also impact the levels of pro-inflammatory mediators, such as IL1β, IL18, and inflammatory lipids, that can reach the lamina propria to recruit neutrophils and induce inflammation. In fact, at 18 h p.i., we did observe reduced numbers of neutrophils and a delayed onset of inflammation in GSDMD-deficient mice in vivo, which may be a result of reduced exposure of the lamina propria to IEC-derived pro-inflammatory cytokines. Notably, another recent study showed that goblet cells rely on GSDMD to secrete mucus on top of the epithelium, and earlier work had established that this may shield against microbes (22, 57, 58). However, in the unperturbed gut, we did not observe any change in the steady-state level of inflammatory marker genes in the cecum tissue of *GsdmD*^−/−^ mice. This is in line with that the mucus covers only the bottom of the crypts of the murine cecum epithelium (which is the main site of *S*.Tm attack in the gut of streptomycin-pretreated mice), while the epithelial cells at the tip of the crypts are lacking such mucus cover, as observed in earlier work on wild type mice (57). In either case, our combined results highlight how epithelial and immune cell GSDMD complement each other in the defense of the mucosal tissue against *S*.Tm invasion.

Recent work in mice has demonstrated that other Gasdermins, e.g., GSDMC and GSDME, also take part in the defense against pathogens and in inflammation (11, 41, 42). In particular, GSDME was shown to induce Caspase-8-driven pyroptosis in neutrophils, which helps to control systemic *Yersinia* infections (11). Additionally, GSDME in epithelial cells promotes inflammation during chemically induced colitis (41). Therefore, it is somewhat surprising that we do not detect any protective effect for GSDME during *S*.Tm infection. GSDME seems to neither influence IEC extrusion efficiency nor to restrict *S*.Tm pathogen loads in the gut tissue, or at systemic sites, not even in a GSDMD-deficient background. Furthermore, GSDMC2 and GSDMC3, which were shown to be highly expressed in epithelial cells of worm-infected mice (42), along with GSDMC1 and GSDMC4 also fail to impact *S*.Tm infection, at least in the streptomycin-pretreated mouse model. The same holds true for GSMDA1-3. Why only GSDMD plays an important protective role during *S*.Tm infection is not fully clear. A reason could be that *S*.Tm is an intracellular pathogen in contrast to worms, and innate immunity against *S*.Tm is consequently dominated by inflammasome signaling. Also, we cannot rule out that *S*.Tm may express yet unidentified virulence factors blocking the action of some Gasdermins. Notably, based on the results from mice lacking all Gasdermins, we were able to answer a long-lasting question in the field and can exclude any redundant protective role among Gasdermins during acute *S*.Tm infection. As GSDMD is the main Gasdermin cleaved by Caspase-1, and as Caspase-1 is an important factor contributing to NAIP/NLRC4-mediated protection of the intestinal mucosa against *S*.Tm, it makes sense that this specific Gasdermin mediates restriction.

In summary, we have assessed the role of Gasdermins in the defense against acute oral *S*.Tm infection of streptomycin-pretreated mice. Our work demonstrates that out of all Gasdermins, only GSDMD exerts a significant and exclusive restrictive function. Both the IEC and lamina propria defense systems notably rely on GSDMD, which contributes to multiple restrictive mechanisms across these two compartments. It remains to be shown whether this is specific for the mouse model used in our experiments and the pathogen strain investigated here, or whether it is of broader relevance for the mucosal defense against other invasive enteropathogenic bacteria.

## Methods

### Bacterial Strains, Plasmids, and Culture Conditions.

All infection experiments were done with *Salmonella* Typhimurium (*S.*Tm) SL1344 (SB300, SmR) if not otherwise specified. Where indicated, *S.*Tm reporter strain harboring the plasmid pM975 (*pssaG*-GFPmut2) was used (49). *S.*Tm was cultured overnight in LB/0.3M NaCl (Sigma Aldrich) with appropriate antibiotics (ca. 12 h) before sub-culturing in 1:20 dilution for 4 h in the same media without antibiotics. For mouse infections, *S*.Tm were washed once and reconstituted with PBS (BioConcepts) before oral gavage. For 3D enteroid infections, *S*.Tm were washed with PBS and reconstituted in DMEM/F-12 (STEMCELL) supplemented with 3% FCS (Thermo Fisher). For 2D enteroid-derived monolayer infections, *S*.Tm were reconstituted in DMEM/F-12 (Gibco) and diluted in complete mouse IntestiCult (STEMCELL) without antibiotics to the desired concentration.

### Mouse Infections.

All mice used were specific pathogen free (SPF) and were maintained in individually ventilated cages of the ETH Zürich mouse facility (EPIC and RCHCI). WT mice were C57BL/6 (congenic marker CD45.2^+^) originally from Charles River (Sulzfeld, Germany). For generation of bone marrow chimeras, B6.SJL-Ptprc^a^ Pepc^b^ mice (congenic marker CD45.1^+^) were used as WT where indicated. All genetic modified mice were of C57BL/6 background. Specifically, the following mouse lines were used: *Nlrc4^−/−^* (B6.C2-Nlrc4tm1Vmd, ref. 59), *GsdmACDE^−/−^* (C57BL/6J- *Gsdma1-3^em1Broz^* x *Gsdmc1-4^em1Broz^* x *Gsdmd^em1Broz^* x *Gsdme^em1Broz^*, this study), *GsdmD^−/−^* (9), *GsdmD*_fsX*^−/−^* (C57BL/6J-*Gsdmd^em1Broz^*, ref. 60), *GsdmD^D88A/D88A^* (C57BL/6J-*Gsdmd^em2Broz^*, ref. 60)*, GsdmE^−/−^* (C57BL/6J-*Gsdme^em1Broz^*, ref. 60), *GsdmD^−/−^GsdmE^−/−^* (C57BL/6J-*Gsdmd^em1Broz^Gsdme^em1Broz^*, ref. 11), *GsdmA^−/−^* (C57BL/6J-*Gsdma1-3^em1Broz^*, this study)*, GsdmC^−/−^* (C57BL/6J-*Gsdmc1-4^em1Broz^,* this study), *ActRFP* (B6.Cg-Tg[CAG-DsRed*MST]1Nagy/J, ref. 61), *IL18^−/−^* (B6.129P2-Il18tm1Aki, ref. 62), *GsdmD^Δ/ΔIEC^* [B6.SJL-Tg(VilCre)997Gum/J (63) x C57BL/6J-*Gsdmd^em3Broz^*, this study]. Genotyping of mice was done by PCR or sequencing. Heterozygous littermates were used as control animals except for *GsdmACDE^−/−^* mice. *GsdmACDE^−/−^* mice were co-housed with WT or *GsdmD^−/−^* mice for at least 2 wk prior to infection. In general, 8- to 15-wk-old mice were infected according to the streptomycin mouse model (64). Briefly, mice were orally pretreated with 25 mg streptomycin sulfate (Sm, AppliChem) 1 d before infection with ~5 × 10^7^ CFU *S.*Tm by oral gavage. Mice were monitored daily, and organs were harvested at the indicated time points. Organs were homogenized in PBS containing 0.5% tergitol and 0.5% BSA using a tissue lyser (Qiagen) and plated on MacConkey agar (Oxoid) with Sm. Cecum tissue was first washed in PBS, incubated for 30 to 60 min in PBS/400 µg/mL gentamycin and washed extensively (6×) in PBS before plating. To generate bone marrow chimeras, mice were gamma-irradiated (1,000 Rad) and 5 × 10^6^ bone marrow cells from the respective mouse line were transferred via the tail vein. Mice received Borgal (Vererinaria AG) in the drinking water for 3 wk and kept at least for 6 wk before infection. Transfer efficiency was measured by flow cytometry. Briefly, the spleen was pressed through a 40 µm cell strainer, and cells were collected in ice-cold PBS. Cells were pelleted (600 g, 5 min, 4 °C) and incubated in BD Cytofix/Cytoperm (BD Biosciences) for 15 min. Fixation was stopped by adding PBS, and cells were washed once with PBS before staining. For staining, cells were incubated in FACS buffer containing CD45.1-Pacific Blue (Biolegend; A20; 1:100), CD45.2-PerCP (Biolegend; 104;1:100), Mouse BD Fc Block (BD Biosciences; 2.4G2; 1:100) for 30 min. After staining, cells were washed two times in FACS buffer and filtered before acquisition. For intravenous infections, 10^4^ *S*.Tm in 100 µL PBS from an overnight or 4 h subculture were injected in the tail vein. For IL18 or IL1β depletion experiments, 200 µg/mouse anti-IL18 (BioXCell, YIGIF74-1G7) or anti- IL1β (BioXCell, B122), respectively, was injected intraperitoneal on the day of pretreatment and infection. For neutrophil depletion experiments, 500 µg/mouse anti-Ly6G (BioXCell, 1A8) was injected intraperitoneal daily starting at pretreatment. For macrophage depletion, 1,000 µg/mouse anti-CSFR1 (BioXCell, AFS98) was injected intraperitoneal 4 d prior infection and 300 µg/mouse every following day until harvesting. All animal experiments were approved by the Kantonales Veterinäramt Zürich (licences 193/2016, 158/2019, and 108/2022).

### Generation of Conditional *GsdmD*, *GsdmA^−/−^*, *GsdmC^−/−^*, and *GsdmACDE^−/−^* Knockout Mice.

To generate conditional *GsdmD* knockout mice (C57BL/6J-*Gsdmd^em3Broz^*), loxP sites were inserted up and downstream of exon 2 of *GsdmD* using the 2 following gRNAs (1: TCCACGGGTTCTATAGACGG’TGG and 2: TCTACTACTCCACTCCTCTG’GGG). To generate *GsdmA*^−/−^ knockout mice (C57BL/6J-*Gsdma1-3^em1Broz^*), the complete *GsdmA1-3* locus was deleted by using the 2 following gRNAs (3: TACGGGGTGGATCACTGCTC’TGG and 4: ACGAGGCTTGGCGGCAGCAA’GGG). To generate *GsdmC^−/−^* knockout mice (C57BL/6J-*Gsdmc1-4^em1Broz^*), the complete *GsdmC1-4* locus was deleted by using the 2 following gRNAs (5: GGCTTGAGTATCAACGCACG’TGGand 6:AGTGTGTGCAATATGGACGG’AGG). To generate *GsdmACDE^−/−^* knockout mice (C57BL/6J- *Gsdma1-3^em1Broz^* x *Gsdmc1-4^em1Broz^* x *Gsdmd^em1Broz^* x *Gsdme^em1Broz^*), *GsdmD^−/−^GsdmE^−/−^* (C57BL/6J-*Gsdmd^em1Broz^Gsdme^em1Broz^*, ref. 11) embryos were injected with gRNAs 3 and 4 or 5 and 6, respectively, and then crossed with each other to reach to the desired genotype. Injection of the gRNAs and Cas9 protein into C57BL/6 embryos was done as described before (65). Biopsies for genotyping were taken at an age of 10 to 12 d. DNA extraction was performed using the KAPA HotStart Mouse Genotyping Kit according to the manufacturer’s protocol.

Genotyping PCR was done using Q5 Polymerase (NEB). For the conditional *GsdmD* knockout mice 3 PCR reactions with 2 primers sets were carried out: PCR-A (CGCTTCCCTTACCTTGAGCA, GTGTCTAGGTGGTTGTGGGG) covering the binding site of gRNA1, PCR-B (CAGCCCTACTTGCTCTAGCC, AGCCAAAACACTCCGGTTCT) covering the binding site of gRNA2. The expected fragment sizes were 339 bp for PCR-A and 388 bp for PCR-B in animals harboring a WT allele, and 373 bp and 423 bp in mice harboring the loxP insertions. After crossing to cre-deleter mice, deletion of exon 2 was verified by PCR using primers CAGCCCTACTTGCTCTAGCC and GTGTCTAGGTGGTTGTGGGG). Genotyping of *GsdmA^−/−^* mice was done carrying out 3 PCR reactions: PCR-WT1-A (TTCAGGATCCCAGTAACCCA, GAGGGGGTGAGTAGGGAGTT) covering the binding site of gRNA3, PCR-WT2-A (GCACACCTTTCTGTTTGGCA, AACTGGAGTTACAGGCAGTC) covering the binding site of gRNA4, and PCR-KO-A (CCTGGCAGCTACAGAAGGTC, ACCACTGCCTGGCTCTTTTT). The expected fragment sizes were 500 bp for PCR-WT1-A, 430 bp for PCR-WT2-A, and 628 bp for PCR-KO-A. Genotyping of *GsdmC^−/−^* mice was done carrying out 3 PCR reactions: PCR-WT1-C (TTCCACGGGTACTTGCTCAG, CCAGGCAGTATGTTCAGGGG) covering the binding site of gRNA5, PCR-WT2-C (GCCCCTGAACCTATCAAGCA, GAGTTGGTGCTGCTTTGGTG) covering the binding site of gRNA6, and PCR-KO-C (TTCCACGGGTACTTGCTCAG, GAGTTGGTGCTGCTTTGGTG). The expected fragment sizes were 378 bp for PCR-WT1-C, 624 bp for PCR-WT2-C, and 439 bp for PCR-KO-C.

### Murine 3D Intestinal Epithelial Enteroids and Infections.

Murine jejunal epithelial enteroids were established and maintained as previously described (31). Briefly, 2 mm pieces of the mouse jejunum were washed in ice-cold PBS, incubated in Gentle cell dissociation reagent (STEMCELL) while rocking (20 rpm, 15 min, RT), and transferred to PBS/0.1% BSA (Chemie Brunschwig AG) to extract intestinal crypts by mechanical shearing. Extracted crypts were filtered through a 70-µm cell strainer, washed, and embedded in 50 µL Matrigel (Chemie Brunschwig AG) domes. The enteroids were maintained in complete mouse IntestiCult medium (STEMCELL) supplemented with PenStrep (Gibco) (37 °C, 5% CO_2_). The culture medium was exchanged every 2 to 4 d. Every 5 to 7 d, the cultures were split by mechanical shearing in RT Gentle dissociation reagent, and the enteroids were re-embedded in 50 µL Matrigel domes (splitting ratio 1:4 to 1:6). Stable enteroid cultures were cryopreserved and thawed for experimentation. *S*.Tm bulk infections were performed after at least 2 wk of culture maintenance. To this end, domes containing ~100 enteroids were dissolved in ice-cold DMEM/F12/3% FCS by pipetting carefully up and down. The enteroids were pelleted by centrifugation (300 g, 5 min, 4 °C), and re-suspended in pre-warmed DMEM/F12/3% FCS without antibiotics. For western blotting, enteroids were infected with *S*.Tm at an estimated MOI of 100 for the indicated durations ((37 °C, 5% CO_2_) assumption of ~1,000 epithelial cells per enteroid (31)). As a positive control, enteroids were treated with 10 µg/mL protective antigen (PA) and 5 µg/mL RodTox (LFn-Rod; Invivogen) in DMEM/F12/3% FCS for 2 h. PA was purified as described previously (40). After 2 h, the enteroids were pelleted by centrifugation (300 g, 5 min, 4 °C) and washed with cold PBS. The washed samples were centrifuged (1,000 g, 5 min, 4 °C), the pellets were snap frozen in liquid nitrogen, and kept at −80 °C until western blotting. For microscopy analysis, *S*.Tm harboring a *pssaG-GFP* reporter was used to infect the enteroids at an estimated MOI of 100 for 40 min (37 °C, 5% CO_2_). After infection, RT DMEM/F12/3% FCS containing 100 μg/mL gentamycin (AppliChem) was added for 15 min (37 °C, 5% CO_2_) to kill extracellular bacteria. The enteroids were pelleted (300 g, 5 min, 4 °C), resuspended in complete IntestiCult supplemented with 25 μg/mL gentamycin and seeded in 25 μL Matrigel domes in prewarmed 8-well glass chamber slides (Thermo Scientific). Domes were solidified for 10 min (37 °C, 5% CO_2_), RT complete IntestiCult containing 25 μg/mL was added, and enteroids were incubated (37 °C, 5% CO_2_) until fixation with 4% paraformaldehyde (PFA; Sigma Aldrich) at 4 h p.i. After fixation, samples were washed three times with PBS, permeabilized with PBS/0.5% Tx-100 (Sigma Aldrich) for ≥10 min, blocked with PBS/10% Normal Goat Serum (NGS) for ≥30 min, and incubated for ≥40 min with TRITC-conjugated Phalloidin (Fluoprobes) and DAPI (Sigma Aldrich). Stained enteroids were extensively washed with PBS and ddH_2_O, the chambers were carefully removed from the glass slides, and the samples were covered with a glass slip using one drop of Mowiol (VWR International AG) per dome.

### Establishment and Infection of 2D Murine Enteroid-Derived Monolayers.

2D murine enteroid-derived monolayers were established as previously described (40). In brief, enteroids were split as described above and cultured in freshly prepared CV medium, i.e., complete mouse IntestiCult supplemented with 3 µM CHIR99021 (Cayman Chemicals) and 1 mM valproic acid (Cayman Chemicals) for 1 wk. The medium was exchanged for fresh CV medium every 2 to 3 d. Glass-bottom 8-well chamber slides (Cellvis) were pre-coated with 75 µg/mL Poly-L-Lysine (Sigma Aldrich) at RT overnight and washed three times with PBS (Gibco). Chamber slides were then dried for 2 h before a 1 mg/mL collagen (Corning) solution in collagen neutralization buffer (20 mM HEPES/53 mM sodium bicarbonate/sodium hydroxide equimolar to acetic acid from the collagen stock) was added to the wells, and the hydrogels were left to solidify for 1 h at 37 °C as previously described (66). CV-pre-treated enteroids were dissociated by mechanical shearing and incubation in Gentle cell dissociation reagent (STEMCELL) for 10 min, nutating at RT. After washing in ice-cold DMEM/F12 (Gibco)/0.25% BSA (Gibco), enteroids were reconstituted in ice-cold DMEM/F12/0.25% BSA and passed through a pre-wetted G25 needle approximately 10 times for mechanical dissociation. Finally, the cell suspension was reconstituted in RT CV medium/10 µM Y-27632 (Sigma Aldrich), and 150,000 cells/cm^2^ were added to the prepared collagen I hydrogels. For chimeric monolayer establishment, equal numbers of cells from each genotype (VilRFP and *GsdmD^−/−^*) were mixed prior to seeding. Monolayers were maintained at 37 °C, 5% CO_2_; and 24 h after establishment, they were washed once in pre-warmed DMEM/F12, and the medium was exchanged for complete mouse IntestiCult without Y-27632. Thereafter, the medium was exchanged for fresh complete mouse IntestiCult every 1 to 2 d. Monolayer infections were performed 72 to 96 h post establishment. Prior to infection, the monolayers were washed once with pre-warmed DMEM/F12 and complete IntestiCult without antibiotics containing 1.5 µM Draq7 was added to each well. After placing the chamber slide in the pre-warmed microscope chamber (37 °C, 5% CO_2_), the prepared *S*.Tm inoculum was added at an MOI of 0.5 to 2 and imaging was started immediately.

### Time-Lapse Imaging of 2D Murine Enteroid-Derived Monolayers.

Time-lapse imaging of 2D murine enteroid-derived monolayers was performed on a custom-built microscope based on an Eclipse Ti2 body (Nikon) with 60×, 0.7 numerical aperture Plan Apo Lambda air and 40×/0.6 Plan Apo Lambda air objectives (Nikon) and a back-lit sCMOS camera (pixel size 11 µm, Prime 95B; Photometrics). Samples were maintained at 37 °C, 5% CO_2_ in a moisturized chamber during imaging. Bright-field imaging was performed using differential interference contrast (DIC), and fluorescence was acquired with an excitation light engine Spectra-X (Lumencor) and emission collection through a quadruple bandpass filter (89402; Chroma). Infected monolayers were imaged at 2 min intervals for a total of 120 min. To quantify IEC permeabilization in response to infection, images were thresholded in Fiji (a version of ImageJ; ref. 67) using the same threshold value for all time-lapse movies from the same experiment, and the area above threshold was enumerated.

### Immunofluorescence Staining and Wide-Field and Confocal Microscopy.

Upon harvesting, mouse cecum tissue was fixed in 4% PFA, saturated in 20% sucrose, and submerged in Optimal Cutting Temperature compound (OCT, Tissue-Tek) before flash freezing in liquid nitrogen. Samples were kept at −80 °C until further analysis. Ceca were cut in 10- to 20-µm thick cross-sections and mounted on glass slides (Superfrost++, Thermo Scientific). Air-dried sections were rehydrated with PBS, permeabilized with PBS/0.5% Tx-100 (Sigma Aldrich), and incubated with PBS/10% Normal Goat Serum (NGS; Reactolab SA) before fluorescence staining. For fluorescence staining the following primary/secondary antibodies and dyes diluted in PBS/10% NGS were used: α-EpCam/CD326 (clone G8.8, Biolegend), α-cleaved Caspase 3 (#9661, Cell Signaling Technology), α-*S*.Tm LPS (O-antigen group B factor 4-5, Difco), α-ICAM-1/CD54 (clone 3E2, BD Biosciences), α-ASC (N-15, Santa Cruz Biotechnology), α-Ly6B.2 (clone 7/4, Bio-Rad), α-rabbit-AlexaFluor488 (Abcam Biochemicals), α-rabbit-Cy3 (Bethyl Laboratories), α-rat-FITC (Jackson), α-rat-Cy3 (Jackson), α-rat-Cy5 (Jackson), α-hamster-Cy5 (Jackson), CruzFluor488-conjugated Phalloidin (Santa Cruz Biotechnology), TRITC-conjugated Phalloidin (Fluoprobes), AlexaFluor647-conjugated Phalloidin (Molecular Probes), and DAPI (Sigma Aldrich). Mowiol (VWR International AG) was used to cover the stained sections with a coverslip. Microscopy was performed using a Zeiss Axiovert 200 m microscope with 10 to 100× objectives, a spinning disc confocal lased unit (Visitron), and an Evolve 512 EMCCD camera (Photometrics). Images were processed or analyzed with Visiview (Visitron) and/or ImageJ. Microscopy quantification was done manually and blindly on at least 2 sections per mouse as previously described (31).

### Flow Cytometric Analysis of Lamina Propria Cells.

Cecum lamina propria cells were isolated and stained as previously described (33). For cell surface staining, cells were incubated in 1 µg/sample Mouse BD Fc Block (BD Biosciences) in 75 µL 10% Brilliant stain buffer (BD Biosciences)/FACS buffer for 5 min at 4 °C prior to adding 25 µL of antibody mix in 10% Brilliant stain buffer/FACS buffer. The following antibodies and dyes were used: CD45-PerCP (Biolegend; 30-F11; 1:100), CD45-BUV563 (BD Biosciences; 30-F11; 1:100), MHCII-BV421 (Biolegend; M5/114.15.2; 1:100), CD11c-PE/Cy7 (Biolegend; N418; 1:200), CD3-BV711 (Biolegend; 145-2C11; 1:200), NK1.1-BV711 (Biolegend; PK136; 1:200), B220-BV711 (Biolegend; RA3-6B2; 1:200), Siglec-F-APC/Cy7 (BD Biosciences; E50-2440; 1:200), Ly-6G-BV650 (Biolegend; 1A8; 1:100), Ly6C-AF700 (Biolegend; HK1.4; 1:200), CD64-PE/Dazzle (Biolegend; X54-5/7.1; 1:100), LIVE/DEAD Fixable Aqua Dead Cell Stain (Life Technologies; 1:1,000). For the intracellular *S*.Tm-LPS staining using α-*S*.Tm LPS (O-antigen group B factor 4-5, Difco) and α-rabbit-AlexaFluor647 (Abcam Biochemicals), cells were incubated with IC fixation buffer (eBioscience, 00-8222) and permeabilization buffer (eBioscience, 00-8333). To set the gates for *S*.Tm-LPS^+^ cells, lamina propria cells were only stained with secondary antibody. Samples were measured on a LSRII (BD Biosciences) or LSR Fortessa (BD Biosciences), and data were analyzed with FlowJo V10 (TreeStar).

### Histology.

For histology analysis, cecum tissue embedded in OCT was snap frozen in liquid nitrogen, cut into 5-µm sections, air-dried, and stained with hematoxylin and eosin. The histology score was determined blindly as described previously (64).

### Lipocalin-2 and TNF ELISA.

Feces or cecum content was used for Lipocalin-2 ELISA and ca. a 5-mm piece of extensively washed cecum tissue for TNF ELISA. The cecum tissue sample was homogenized in PBS/0.5% Tergitol/0.5% BSA (Sigma Aldrich, Chemie Brunschwig AG) supplemented with protease inhibitor cocktail (Roche). Lipocalin-2 (R&D Systems) and high-sensitivity TNF (Invitrogen) ELISA was performed according to the manufacturer’s protocols.

### RT-qPCR.

Cecum tissue samples were snap frozen in RNAlater (Invitrogen) and kept at −80 °C. The RNeasy Mini Kit (Qiagen) was used to isolate RNA and the RT^2^ HT First Strand cDNA Kit (Qiagen) to reverse transcribe to cDNA. A QuantStudio 7 Flex StepOne Plus Cycler was used to perform qPCR analysis with FastStart Universal SYBR Green Master reagents (Roche). Only validated primers from Qiagen were used.

### Western Blotting.

Cecum tissue samples were homogenized in ice-cold PBS (Thermofisher Scientific) supplemented with protease inhibitor cocktail (Merck) for 20 s using an Omni International TH tissue homogenizer. Cells were then lysed in LDS sample buffer (Thermofisher Scientific) + 10 mM DTT and boiled at 98 °C for 10 min before western blotting. The pelleted enteroids (~500 enteroids/sample) were resuspended in 250 µL TrisHCl pH7.4 + 4% SDS supplemented with protease inhibitor cocktail and passed 10 times through a 25-gauge needle. Samples were then diluted in LDS sample buffer +10 mM DTT before boiling at 98 °C for 10 min. Protein extracts were run on SDS-PAGE gels and then blotted onto nitrocellulose membranes (Amersham). Membranes were blocked in 5% milk in TBS + 0.1% Tween20 (TBS-T) and incubated with primary antibodies overnight at 4 °C. Membranes were subsequently washed three times in TBS-T and incubated with secondary antibody for 1 h at RT. Following three further washes in TBS-T, membranes were developed with ECL using an iBright imaging device.

Antibodies used were anti-GSDMD (Abcam ab209845, 1:1,000), Vinculin (Abcam ab91459, 1:1,000), and anti-rabbit HRP (Southern Biotech 4030-05, 1:5,000).

### Statistical Analysis.

The Mann-Whitney *U* test was used to assess statistical significance where applicable as indicated in the figure legends.

## Supplementary Material

Appendix 01 (PDF)

## Data Availability

All study data are included in the article and/or *SI Appendix*.
