# Supplementary material for: Gasdermin D is the only Gasdermin that provides protection against acute Salmonella gut infection in mice
Source: Proc Natl Acad Sci U S A. 2023 Nov 21;120(48):e2315503120. doi: 10.1073/pnas.2315503120 (PMC10691232; doi:10.1073/pnas.2315503120)
Supplement: Supplementary file 1 — Appendix 01 (PDF) [file pnas.2315503120.sapp.pdf]

Supporting information

Gasdermin D is the only Gasdermin that provides protection against acute *Salmonella* gut infection in mice

Stefan A. Fattinger[1,2,3,†*], Luca Maurer[1,†], Petra Geiser[2], Elliott M. Bernard[4], Ursina Enz[1], Suwannee Ganguillet[1], Ersin Gül[1], Sanne Kroon[1], Benjamin Demarco[4], Vanessa Mack[4], Markus Furter[1], Manja Barthel[1], Pawel Pelczar[5], Feng Shao[6], Petr Broz[4], Mikael E. Sellin[2*], Wolf-Dietrich Hardt[1*]

Affiliations:
[1] Institute of Microbiology, Department of Biology, ETH Zurich, Zurich, Switzerland
[2] Science for Life Laboratory, Department of Medical Biochemistry and Microbiology, Uppsala University, Uppsala, Sweden
[3] Division of Immunology and Molecular Medicine, Department of Molecular and Cell Biology, University of California, Berkeley, CA, USA.
[4] Department of Immunobiology, University of Lausanne, Epalinges, Switzerland
[5] Center for Transgenic Models, University of Basel, Basel, Switzerland.
[6] National Institute of Biological Sciences, 102206 Beijing, China

* Correspondence: SAF; sfattinger@berkeley.edu, MES; mikael.sellin@imbim.uu.se, WDH; hardt@micro.biol.ethz.ch

[†] Contributed equally

Figure S1 - Supplementary figures to Fig 1

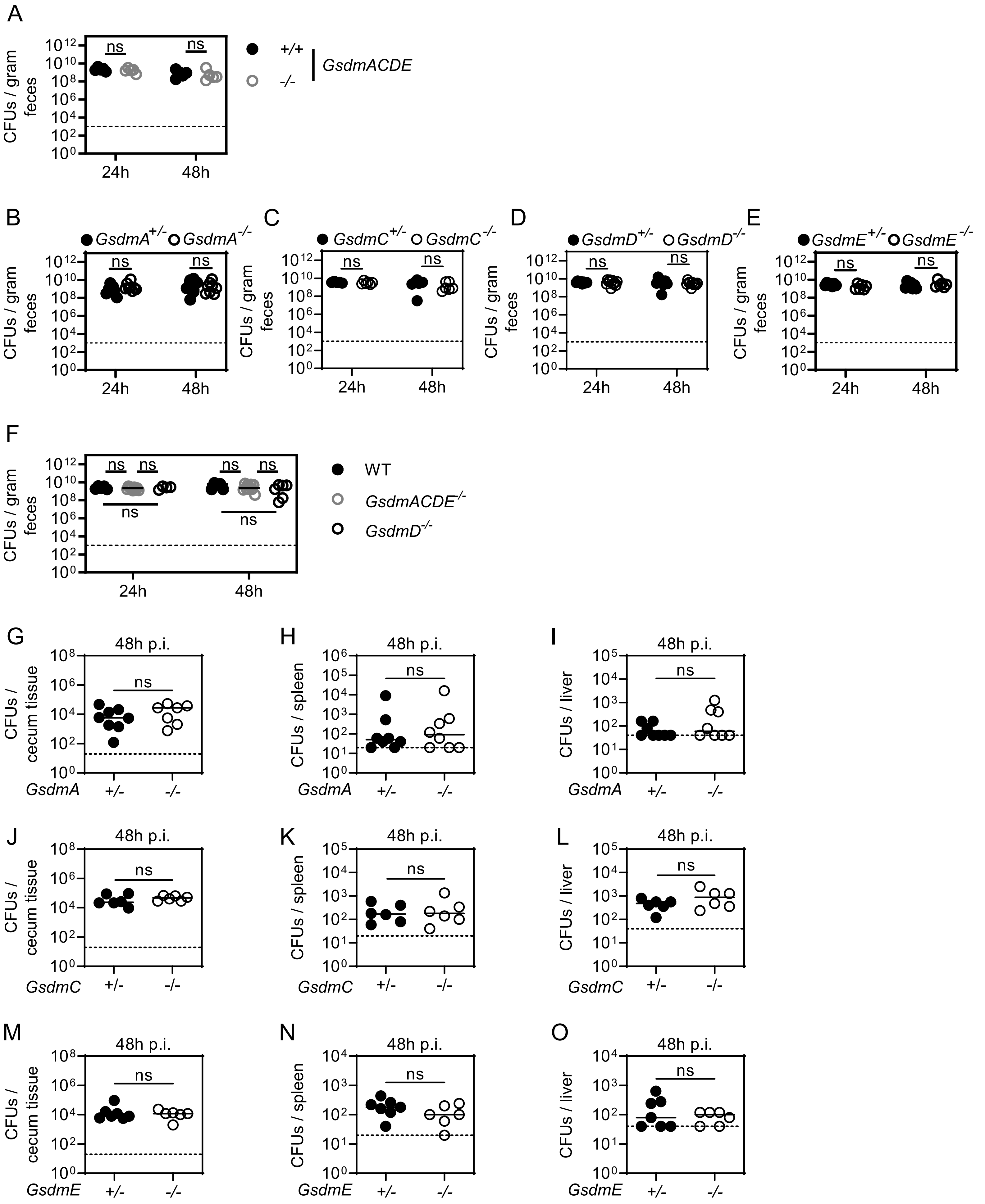

Figure S1. (Supplementary Figure to Fig 1)
(**A**) *S.*Tm pathogen loads in feces of 48h infections with WT and *GsdmACDE*$^{-/-}$ mice over time. (**B-E**) *S.*Tm pathogen loads in feces over time of littermate-controlled 48h infections with (**B**) GSDMA-deficient, (**C**) GSDMC-deficient, (**D**) GSDMD-deficient, and (**E**) GSDME-deficient mice. (**F**) *S.*Tm pathogen loads in feces of 48h infections with WT, *GsdmACDE*$^{-/-}$, and *GsdmD*$^{-/-}$ mice over time. (**G-I**) 48h infection of *GsdmA*$^{+/-}$ and *GsdmA*$^{-/-}$ littermates (belongs to B). *S.*Tm pathogen loads in (**G**) cecum tissue, (**H**) spleen, and (**I**) liver. (**J-L**) 48h infection of *GsdmC*$^{+/-}$ and *GsdmC*$^{-/-}$ littermates (belongs to C). *S.*Tm pathogen loads in (**J**) cecum tissue, (**K**) spleen, and (**L**) liver. (**M-O**) 48h infection of *GsdmE*$^{+/-}$ and *GsdmE*$^{-/-}$ littermates (belongs to E). *S.*Tm pathogen loads in (**M**) cecum tissue, (**N**) spleen, and (**O**) liver. In all panels each data point represents one mouse. ≥5 mice per group from ≥2 independent experiments for each comparison. Line at median. Dotted line represents detection limit. Mann-Whitney U-test (ns - not significant).

Figure S2 - Steady state analysis

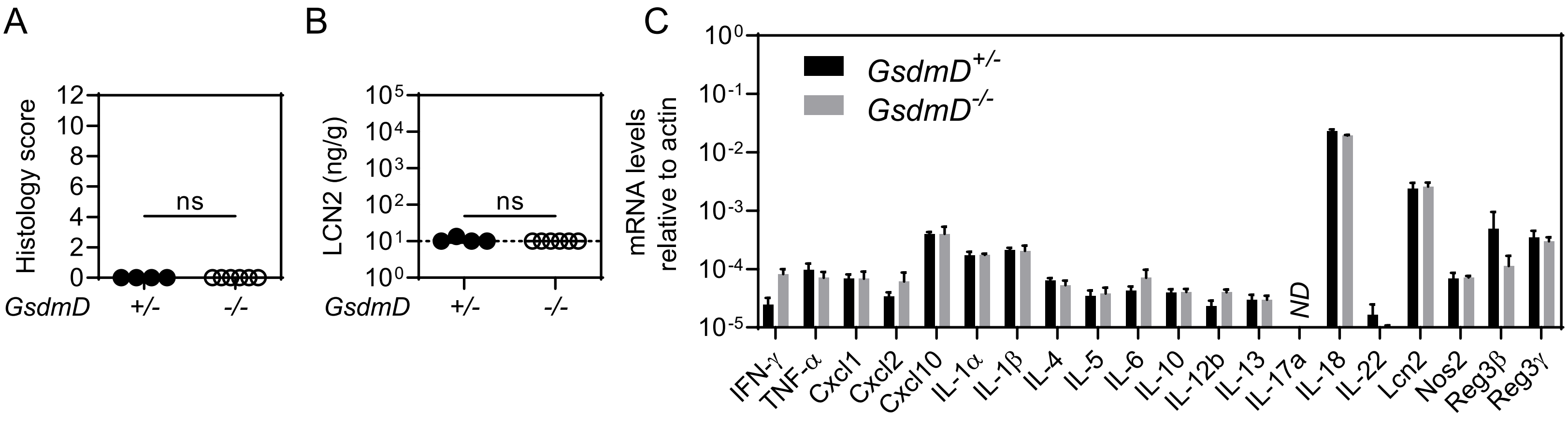

Figure S2. (Steady state analysis of inflammation)
(**A-B**) No detectable inflammatory pre-condition in the gut mucosa of GSDMD-deficient mice. (**A**) Microscopy-based quantification of histology scores from H&E-stained cecum tissue sections. (**B**) Lipocalin-2 levels of feces. In A, B, each data point represents one mouse. Line at median. Dotted line represents detection limit. (**C**) mRNA levels of pro-inflammatory cytokines and anti-microbial peptides. Means with SD are indicated. For all panels ≥4 mice per group. Mann-Whitney U-test (ns - not significant).

Figure S3 - Supplementary figures to Fig 2

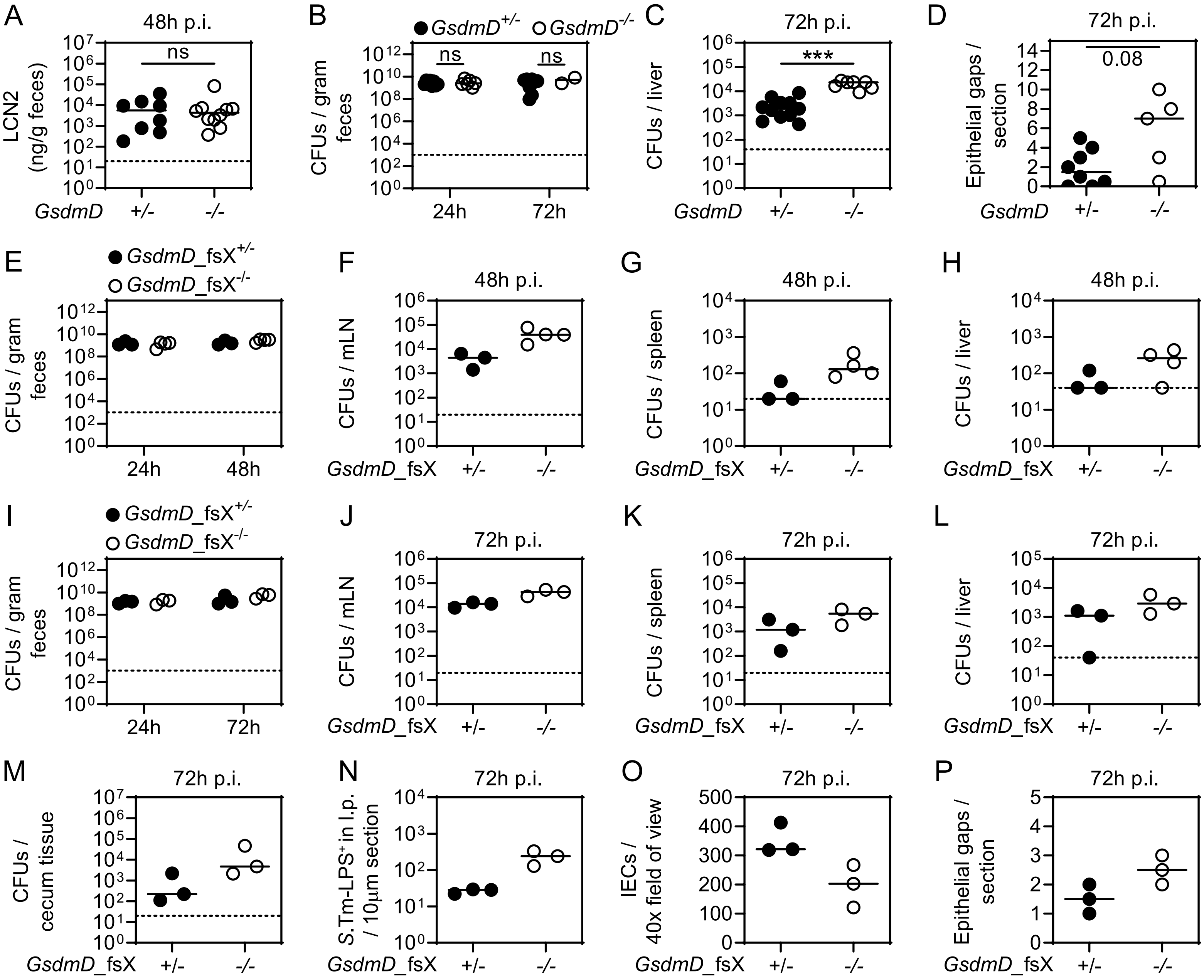

Figure S3. (Supplementary figure to Fig 2)

(**A**) 48h infection of *GsdmD*$^{+/-}$ and *GsdmD*$^{-/-}$ littermates. Lipocalin-2 levels of feces at 48h p.i. (**B-D**) 72h infection of *GsdmD*$^{+/-}$ and *GsdmD*$^{-/-}$ littermates. *S.*Tm CFU pathogen loads in (**B**) feces over time and (**C**) liver. (**D**) Microscopy-based quantification of epithelial gaps in the cecum tissue at 72h p.i. (**E-P**) Independent GSDMD-deficient mouse line (caused by genetic frameshift, *GsdmD_fsX*) exhibits same phenotype. (**E-H**) 48h infection of *GsdmD*_fsX$^{+/-}$ and *GsdmD*_fsX$^{-/-}$ littermates. *S.*Tm pathogen loads in (**E**) feces over time and at 48h p.i. in (**F**) mesenteric lymph nodes, (**G**) spleen, and (**H**) liver. (**I-P**) 72h infection of *GsdmD*_fsX$^{+/-}$ and *GsdmD*_fsX$^{-/-}$ littermates. *S.*Tm pathogen loads in (**I**) feces over time and at 72h p.i. in (**J**) mesenteric lymph nodes, (**K**) spleen, (**L**) liver, and (**M**) cecum tissue. Microscopy-based quantification at 72h p.i. of (**N**) *S.*Tm-LPS^{+} cells in the lamina propria, (**O**) IECs per field of view, and (**P**) epithelial gaps per section. In all panels each data point represents one mouse. ≥3 mice per group. Line at median. Dotted line represents detection limit. Mann-Whitney U-test (ns - not significant, ***p<0.001).

Figure S4 - Supplementary figures to Fig 3

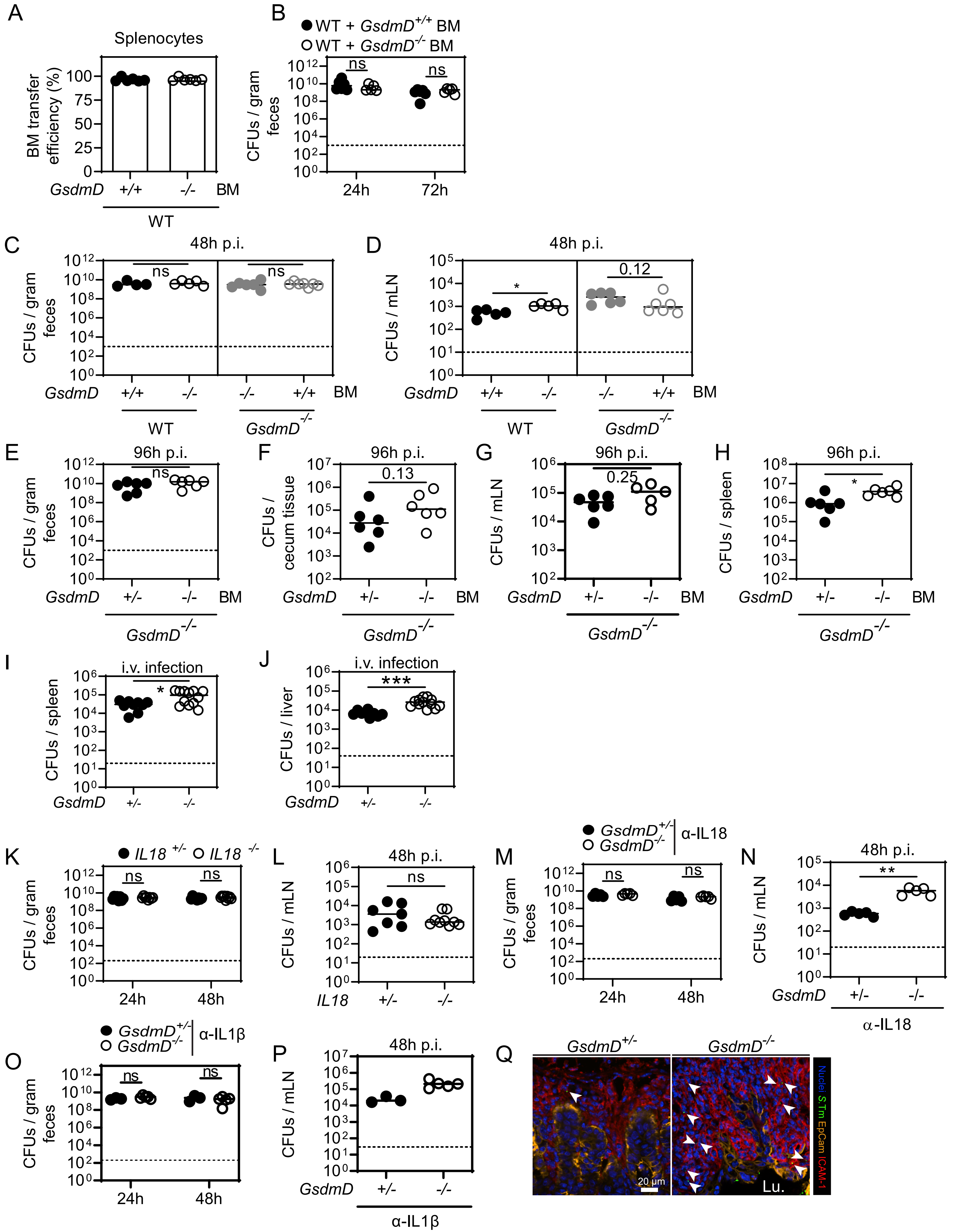

Figure S4. (Supplementary figure to Fig 3)

(**A-B**) 72h infection of BM chimeras. (**A**) BM transfer efficiency for splenocytes. (**B**) *S.*Tm pathogen loads in feces over time. (**C-H**) *GsdmD*$^{-/-}$ BM results in elevated *S.*Tm pathogen loads locally and systemically at 48 and 96h p.i. (**C-D**) 48h infection of BM chimeras with *S.*Tm harboring a *pssaG-GFP* reporter. *S.*Tm pathogen loads in (**C**) feces, and (**D**) mesenteric lymph nodes. (**E-H**) 96h infection of BM chimeras with *S.*Tm. *S.*Tm pathogen loads in (**E**) feces, (**F**) cecum tissue, (**G**) mesenteric lymph nodes, and (**H**) spleen. (**I-J**) GSDMD limits *S.*Tm pathogen loads during systemic *S.*Tm infection. *S.*Tm pathogen loads at 24h p.i. of intravenously infected mice in (**I**) spleen, and (**J**) liver. (**K-L**) At 48h p.i., IL18-deficient mice exhibit similar *S.*Tm pathogen loads in (**K**) feces, (**L**) mesenteric lymph nodes. (**M-N**) IL18 is dispensable for GSDMD-dependent *S.*Tm restriction. *S.*Tm pathogen loads at 48h p.i. (**M**) in feces, and (**N**) in mesenteric lymph nodes of GsdmD$^{+/-}$ and GsdmD$^{-/-}$ littermates in the presence of a neutralizing IL18 antibody. Note, this infection was performed with *S.*Tm harboring a *pssaG-GFP* reporter which explains the overall ca. 10x lower pathogen loads. (**O-P**) IL1β is dispensable for GSDMD-dependent *S.*Tm restriction. *S.*Tm pathogen loads (**O**) in feces over time and (**P**) in mesenteric lymph nodes at 48h p.i. of *GsdmD*$^{+/-}$ and *GsdmD*$^{-/-}$ littermates in the presence of a neutralizing IL1β antibody. (**Q**) Representative micrographs of *S.*Tm infected cecum tissue sections at 72h p.i. of *GsdmD*$^{+/-}$ and *GsdmD*$^{-/-}$ littermates, stained for *S.*Tm-LPS (belongs to Fig 3G-H). Arrowheads indicate *S.*Tm in lamina propria. Lu. - Lumen. In A-P, each data point represents one mouse. Line at median. Dotted line represents detection limit. Mann-Whitney U-test (ns - not significant, *p<0.05, **p<0.01, ***p<0.001).

Figure S5 - Supplementary figures to Fig 3

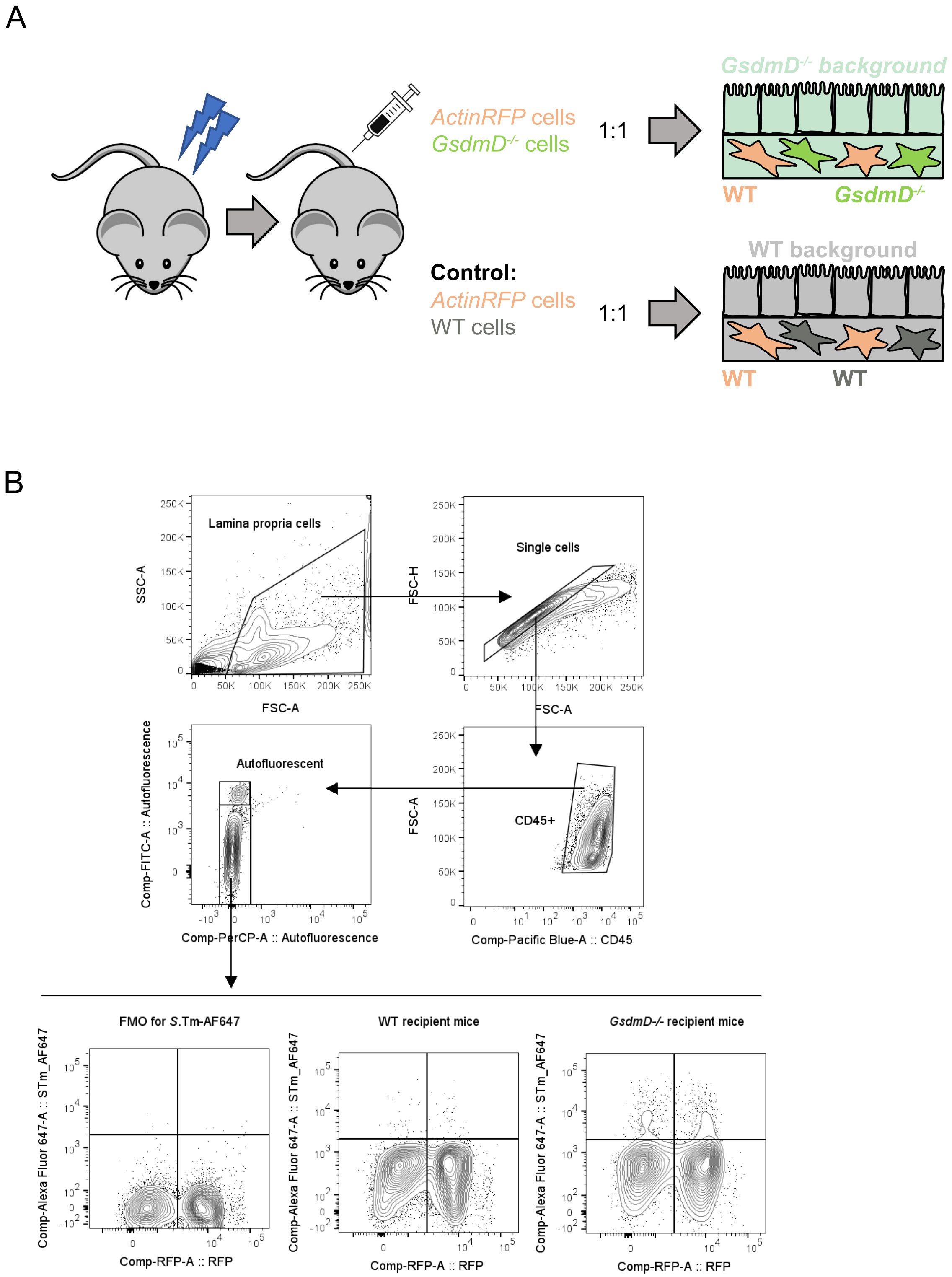

Figure S5. (Supplementary figure to Fig 3)
(**A**) Graphical illustration of experimental setup. (**B**) Flow cytometry gating strategy for Fig 3F

Figure S6 - Supplementary figures to Fig 3

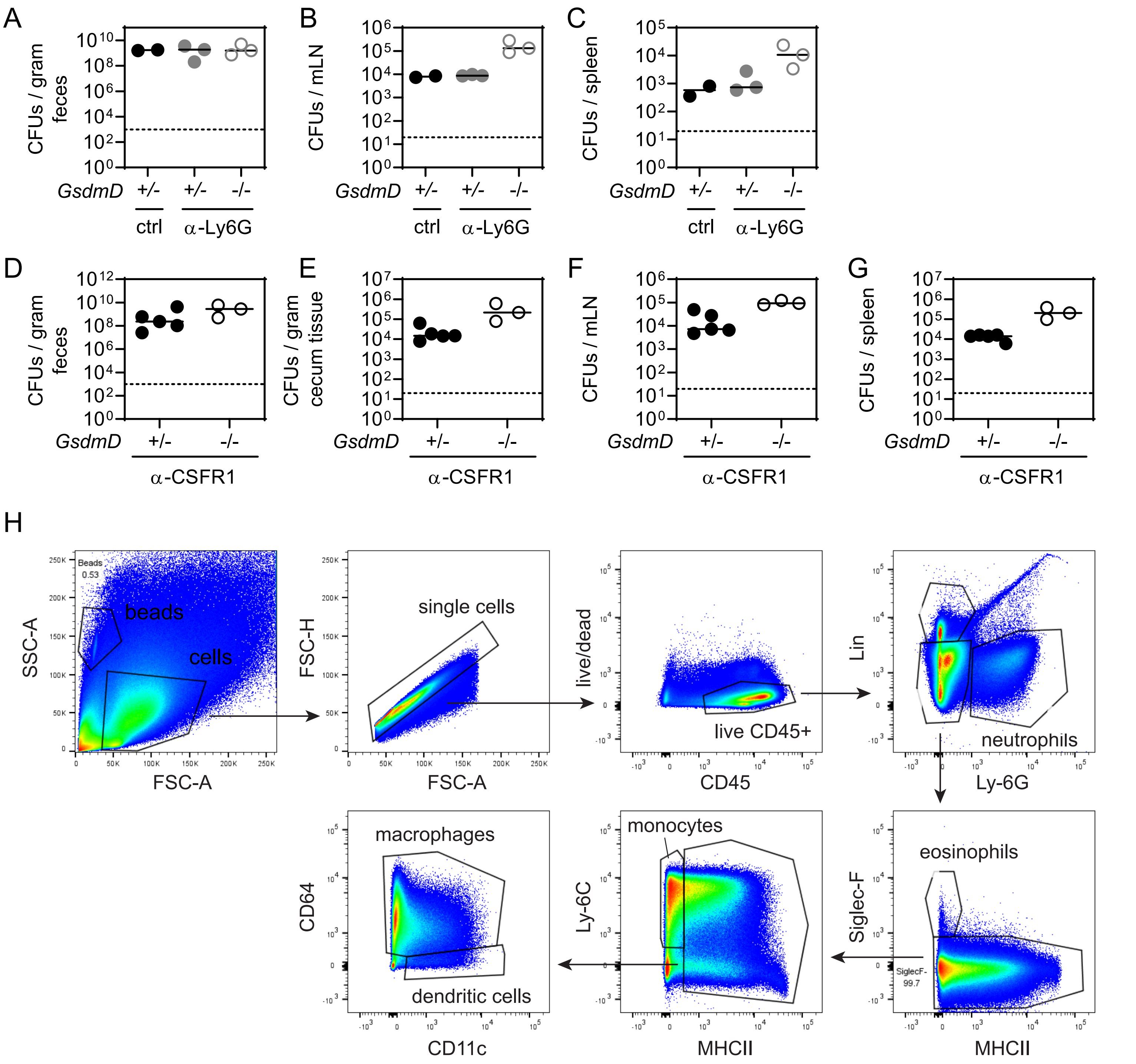

Figure S6. (Supplementary figure to Fig 3)
(**A-G**) Depletion neither of neutrophils, nor macrophages, eliminates the GSDMD-dependent phenotype. (**A-C**) Neutrophil depletion by anti-Ly6G antibody in a 48h *S.*Tm infection of *GsdmD*$^{+/-}$ and *GsdmD*$^{-/-}$ littermates. Control mice were injected with PBS. *S.*Tm pathogen loads in (**A**) feces, (**B**) mesenteric lymph nodes, and (**C**) spleen. (**D-G**) Macrophage depletion by anti-CSFR1 antibody in a 72h *S.*Tm infection of *GsdmD*$^{+/-}$ and *GsdmD*$^{-/-}$ littermates. *S.*Tm pathogen loads in (**D**) feces, (**E**) cecum tissue, (**F**) mesenteric lymph nodes, and (**G**) spleen. In all panels each data point represents one mouse. Line at median. Dotted line represents detection limit. (**H**) Gating strategy of flow cytometry analysis to determine lamina propria cell types in Fig 3I-J

Figure S7 - Supplementary figures to Fig 4

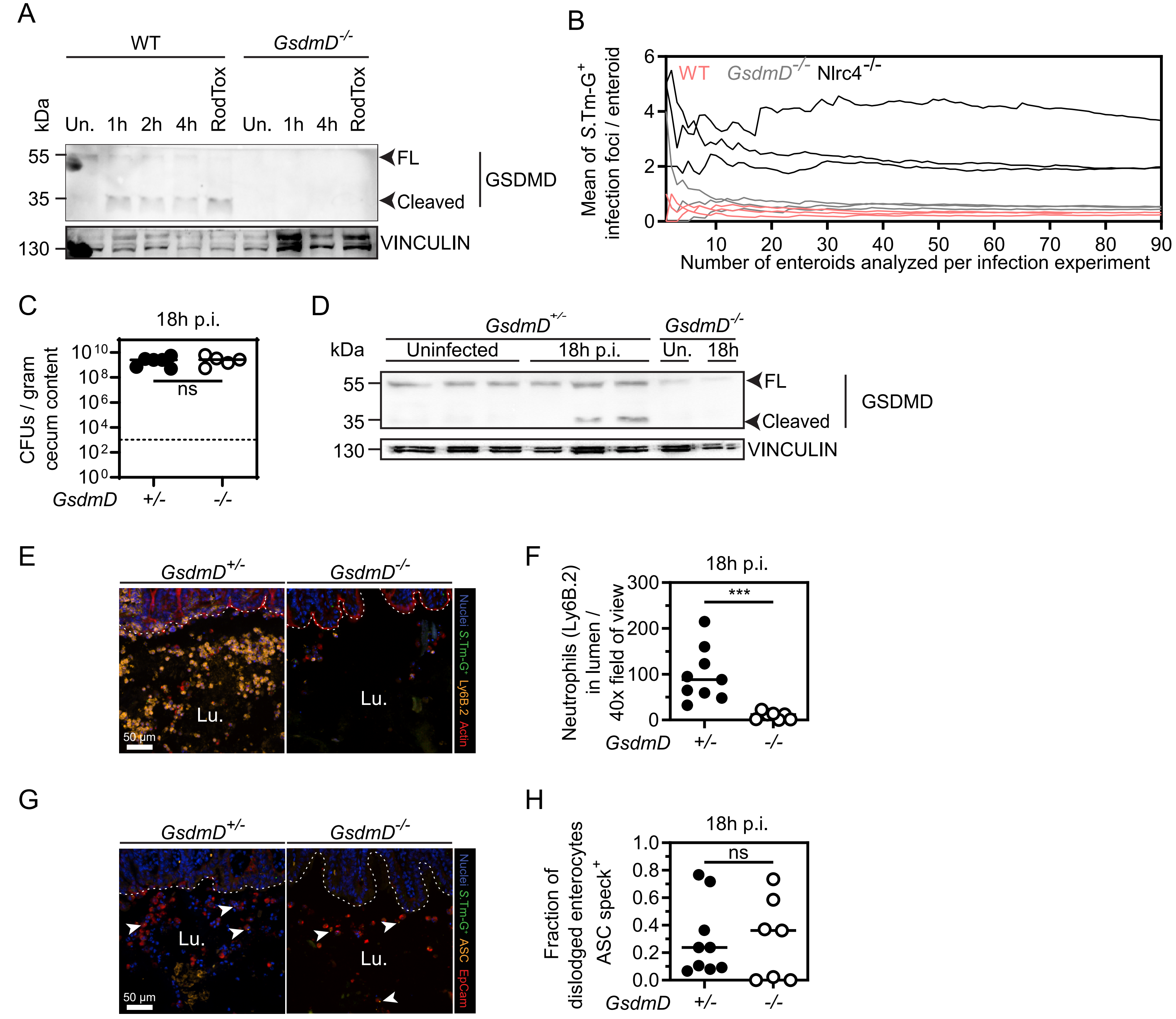

Figure S7. (Supplementary figure to Fig 4)
(**A**) Western blot analysis of WT and *GsdmD*$^{-/-}$ 3D enteroids which were infected in bulk with *S*.Tm for 1h, 2h or 4h. Un. – uninfected. FL – full-length. (**B**) Determination of a sufficient sampling size for Fig 4A-B. Each line represents one replicate infection. 3 replicate infections per genotype. (**C-H**) 18h infection of *GsdmD*$^{+/-}$ and *GsdmD*$^{-/-}$ littermates with *S*.Tm harboring a *pssaG-GFP* reporter. (**C**) *S*.Tm pathogen loads in cecum content. (**D**) Representative western blot of cecum tissue from *GsdmD*$^{+/-}$ and *GsdmD*$^{-/-}$ mice infected with *S*.Tm harboring a *pssaG-GFP* reporter for 18h. Un. – uninfected. FL – full-length. Note, for 4 out of 5 infected mice we could detect GSDMD-cleavage. (**E**) Representative micrographs of cecum tissue sections stained for neutrophils (Ly6B.2^{+}). Lu. - Lumen. (**F**) Microscopy-based quantification of neutrophils in lumen / 40x field of view. (**G**) Representative micrographs of cecum tissue sections stained for ASC specks. Arrowheads indicate ASC speck^{+} dislodged enterocytes. Lu. - Lumen. (**H**) Microscopy-based quantification of the fraction of ASC speck^{+} dislodged enterocytes. In A representative blot from two independent experiments. In C, F, H, each data point represents one mouse. ≥5 mice per group from ≥2 independent experiments for each comparison. Line at median. Dotted line represents detection limit. Mann-Whitney U-test (ns - not significant, ***$p<0.001$).

Figure S8

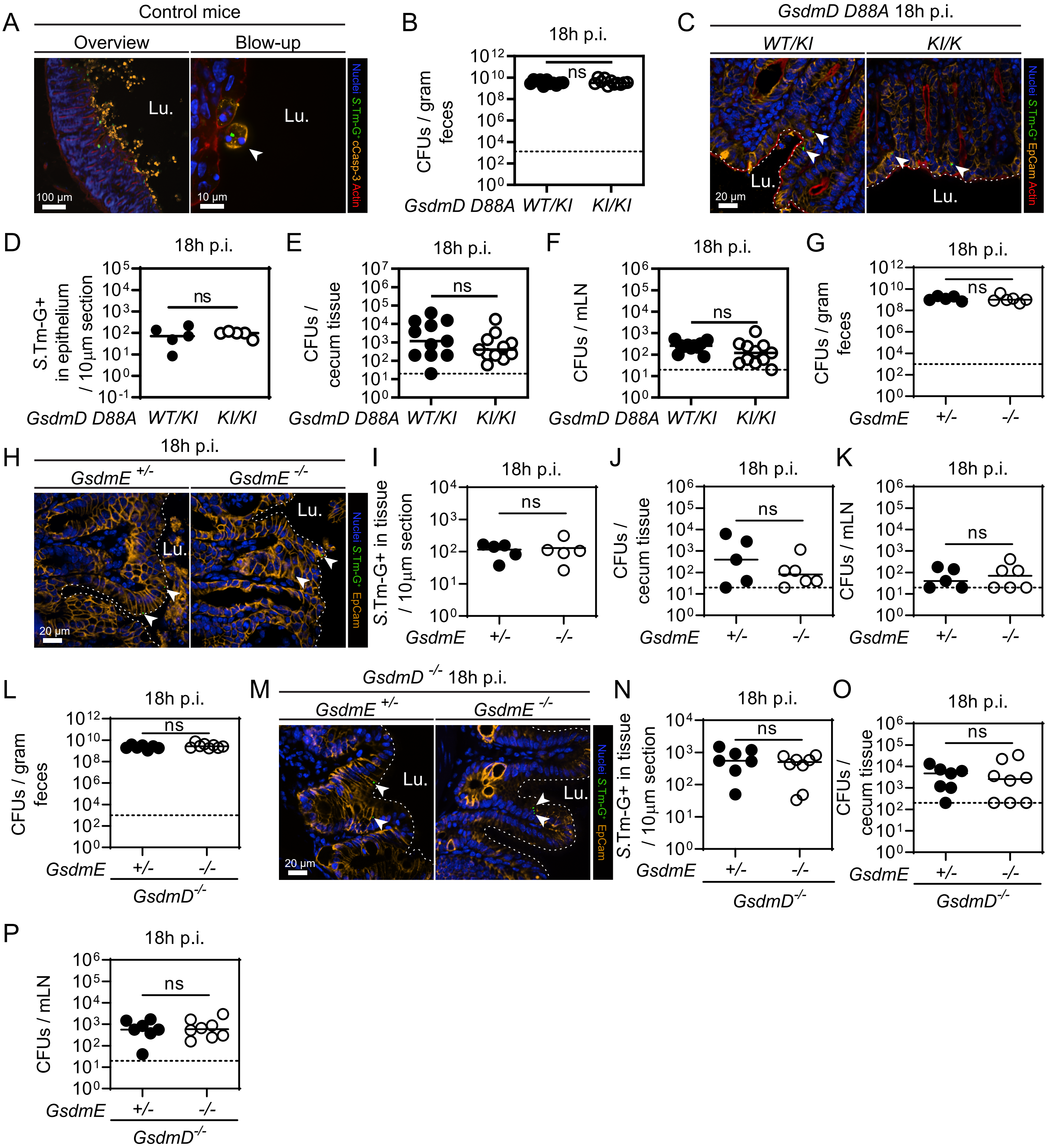
A
Control mice
Overview
Blow-up
Lu.
100 µm
10 µm
Nuclei S.Tm-G+ cCasp-3 Actin
B
18h p.i.
CFUs / gram feces
ns
GsdmD D88A WT/KI KI/KI
C
GsdmD D88A 18h p.i.
WT/KI
KI/K
Lu.
20 µm
Nuclei S.Tm-G+ EpCam Actin
D
18h p.i.
S.Tm-G+ in epithelium / 10µm section
ns
GsdmD D88A WT/KI KI/KI
E
18h p.i.
CFUs / cecum tissue
ns
GsdmD D88A WT/KI KI/KI
F
18h p.i.
CFUs / mLN
ns
GsdmD D88A WT/KI KI/KI
G
18h p.i.
CFUs / gram feces
ns
GsdmE +/- -/-
H
18h p.i.
GsdmE +/-
GsdmE -/-
Lu.
20 µm
Nuclei S.Tm-G+ EpCam
I
18h p.i.
S.Tm-G+ in tissue / 10µm section
ns
GsdmE +/- -/-
J
18h p.i.
CFUs / cecum tissue
ns
GsdmE +/- -/-
K
18h p.i.
CFUs / mLN
ns
GsdmE +/- -/-
L
18h p.i.
CFUs / gram feces
ns
GsdmE +/- -/-
GsdmD-/-
M
GsdmD -/- 18h p.i.
GsdmE +/-
GsdmE -/-
Lu.
20 µm
Nuclei S.Tm-G+ EpCam
N
18h p.i.
S.Tm-G+ in tissue / 10µm section
ns
GsdmE +/- -/-
GsdmD-/-
O
18h p.i.
CFUs / cecum tissue
ns
GsdmE +/- -/-
GsdmD-/-
P
18h p.i.
CFUs / mLN
ns
GsdmE +/- -/-
GsdmD-/-

Figure S8. (Supplementary figure to Fig 4)

(**A**) Representative micrographs of cecum tissue sections from a control mouse at 9h p.i. infected with *S.*Tm harboring a *pssaG-GFP* reporter and stained for cleaved Caspase-3. Arrowhead indicates an infected expelling enterocyte. Lu. - Lumen. (**B-F**) 18h infection of $GsdmD^{WT/D88A}$ (referred as *WT/KI*) and $GsdmD^{D88A/D88A}$ (referred as *KI/KI*) littermates with *S.*Tm harboring a *pssaG-GFP* reporter. (**B**) *S.*Tm pathogen loads in feces. (**C**) Representative micrographs of cecum tissue sections. Arrowheads indicate *S.*Tm-G^+ in epithelium. Lu. - Lumen. (**D**) Microscopy-based quantification of *S.*Tm-G^+ in epithelium. *S.*Tm pathogen loads in (**E**) cecum tissue, and (**F**) mesenteric lymph nodes. (**G-K**) 18h infection of $GsdmE^{+/-}$ and $GsdmE^{-/-}$ littermates infected with *S.*Tm harboring a *pssaG-GFP* reporter. (**G**) *S.*Tm pathogen loads in feces. (**H**) Representative micrographs of cecum tissue sections. Arrowheads indicate *S.*Tm-G^+. Lu. - Lumen. (**I**) Microscopy-based quantification of *S.*Tm-G^+ in cecum tissue. *S.*Tm pathogen loads in (**J**) cecum tissue, and (**K**) mesenteric lymph nodes. (**L-P**) 18h infection of $GsdmD^{-/-}xGsdmE^{+/-}$ and $GsdmD^{-/-}xGsdmE^{-/-}$ littermates infected with *S.*Tm harboring a *pssaG-GFP* reporter. (**L**) *S.*Tm pathogen loads in feces. (**M**) Representative micrographs of cecum tissue sections. Arrowheads indicate *S.*Tm-G^+. Lu. - Lumen. (**N**) Microscopy-based quantification of *S.*Tm-G^+ in cecum tissue. *S.*Tm pathogen loads in (**O**) cecum tissue, and (**P**) mesenteric lymph nodes. In B, D-G, I-L, N-P, each data point represents one mouse. ≥5 mice per group from ≥2 independent experiments for each comparison. Line at median. Dotted line represents detection limit. Mann-Whitney U-test (ns - not significant).

Figure S9 - Supplementary figures to Fig 5

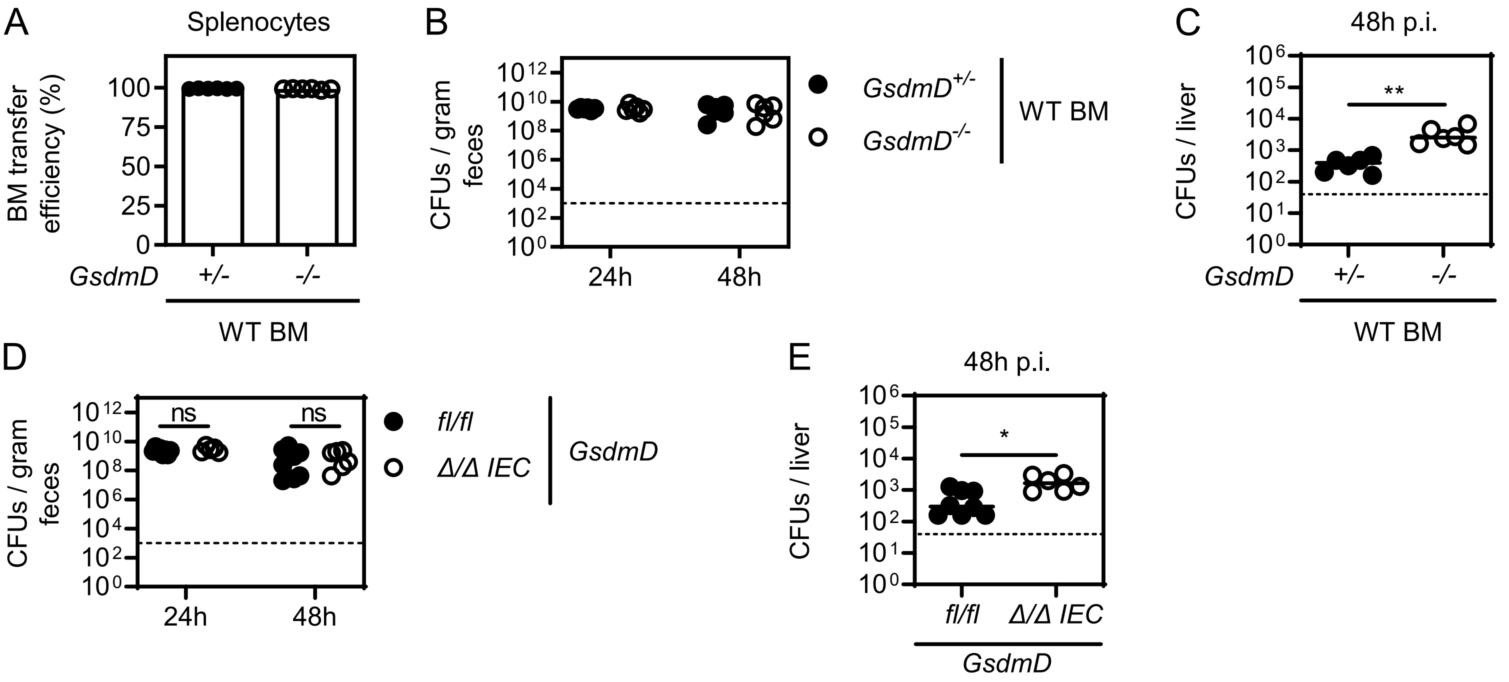

Figure S9. (Supplementary figure to Fig 5)
(**A-C**) 48h infection of BM chimeras. (**A**) BM transfer efficiency for splenocytes. *S.*Tm pathogen loads in (**B**) feces over time and (**C**) liver. (**D-E**) 48h infection of $GsdmD^{fl/fl}$ and $GsdmD^{\Delta/\Delta IEC}$ littermates. *S.*Tm pathogen loads in (**D**) feces over time and (**E**) liver. Each data point represents one mouse. Line at median. Dotted line represents detection limit. Mann-Whitney U-test (ns - not significant, *p<0.05, **p<0.01).
